# Sulfonate-Functionalized Metal–Organic Framework as a Porous “Proton Reservoir” for Boosting Electrochemical Reduction of Nitrate to Ammonia

**DOI:** 10.1021/acsami.4c14786

**Published:** 2024-11-01

**Authors:** Yun-Shan Tsai, Shang-Cheng Yang, Tzu-Hsien Yang, Chung-Huan Wu, Tzu-Chi Lin, Chung-Wei Kung

**Affiliations:** † Department of Chemical Engineering, National Cheng Kung University, 1 University Road, Tainan City 70101, Taiwan; ‡ Program on Key Materials, Academy of Innovative Semiconductor and Sustainable Manufacturing, National Cheng Kung University, 1 University Road, Tainan City 70101, Taiwan

**Keywords:** ammonia production, electrocatalysis, ionic MOF, microenvironment, postsynthetic modification, zirconium-based MOF

## Abstract

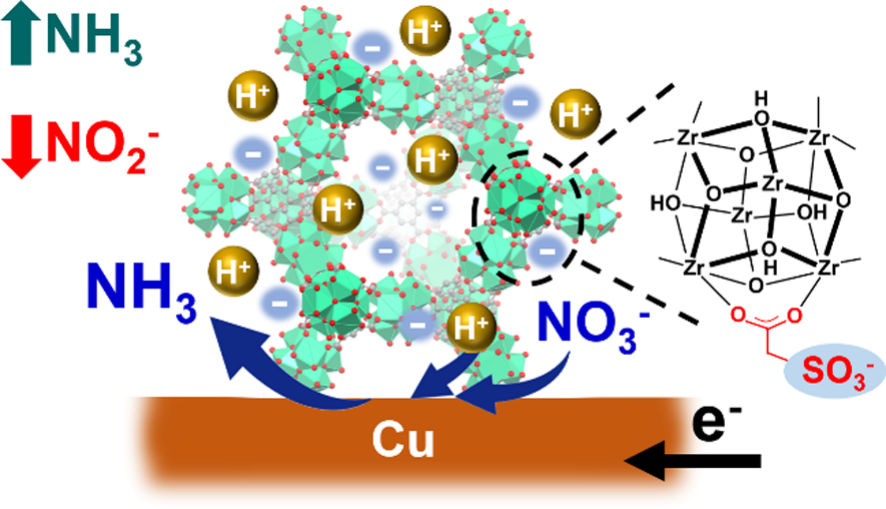

The electrochemical reduction reaction of nitrate (NO_3_RR) is an attractive route to produce ammonia at ambient conditions, but the conversion from nitrate to ammonia, which requires nine protons, has to compete with both the two-proton process of nitrite formation and the hydrogen evolution reaction. Extensive research efforts have thus been made in recent studies to develop electrocatalysts for the NO_3_RR facilitating the production of ammonia. Rather than designing another better electrocatalyst, herein, we synthesize an electrochemically inactive, porous, and chemically robust zirconium-based metal–organic framework (MOF) with enriched intraframework sulfonate groups, SO_3_-MOF-808, as a coating deposited on top of the catalytically active copper-based electrode. Although both the overall reaction rate and electrochemically active surface area of the electrode are barely affected by the MOF coating, with negatively charged sulfonate groups capable of enriching more protons near the electrode surface, the MOF coating significantly promotes the selectivity of the NO_3_RR toward the production of ammonia. In contrast, the use of MOF coating with positively charged trimethylammonium groups to repulse protons strongly facilitates the conversion of nitrate to nitrite, with selectivity of more than 90% at all potentials. Under the optimal operating conditions, the copper electrocatalyst with SO_3_-MOF-808 coating can achieve a Faradaic efficiency of 87.5% for ammonia production, a nitrate-to-ammonia selectivity of 95.6%, and an ammonia production rate of 97 μmol/cm^2^ h, outperforming all of those achieved by both the pristine copper (75.0%; 93.9%; 87 μmol/cm^2^ h) and copper with optimized Nafion coating (83.3%; 86.9%; 64 μmol/cm^2^ h). Findings here suggest the function of MOF as an advanced alternative to the commercially available Nafion to enrich protons near the surface of electrocatalyst for NO_3_RR, and shed light on the potential of utilizing such electrochemically inactive MOF coatings in a range of proton-coupled electrocatalytic reactions.

## 1 Introduction

Electrochemical reduction of nitrogen has been considered an appealing pathway to produce ammonia at a much milder condition compared to the conventionally used energy-intensive Haber–Bosch process.^1,2^ However, the high energy required to break the N ≡ N triple bond as well as the low solubility of nitrogen gas in electrolytes strongly limit the production rate of ammonia through such a pathway.^3,4^ The electrochemical reduction reaction of nitrate (NO_3_RR), which can convert nitrate ions (NO_3_^–^) from wastewater into ammonia at ambient conditions, has thus become a promising approach to producing ammonia at a higher production rate.^5−7^ Several electrocatalysts for NO_3_RR have been developed and investigated in recent years,^8^ and copper (Cu) is in general considered as the most promising non-noble-metal catalyst for NO_3_RR to produce ammonia.^5,6,8−10^ In addition to the generation of ammonia, the formation of unwanted nitrite ions and the competing hydrogen evolution reaction (HER) are two major side reactions occurring on the Cu surface.^5,11,12^ These reactions are listed as follows.
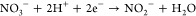
1
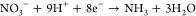
2
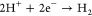
3

Thermodynamic standard potentials of reactions (1) and (2) were reported as +0.01 and −0.12 V vs. standard hydrogen electrode (SHE), respectively,^13^ though much more negative potentials are usually required for both reactions to overcome their high activation energy.

From the eqs 1–3, it could be found that the conversion of nitrate into ammonia requires nine protons, much more than that required for producing nitrite. Thus, although a high concentration of protons may be beneficial for HER, it could also remarkably boost the selectivity toward ammonia production against the formation of nitrite, which is highly desirable for NO_3_RR. Thus, modulating the local concentration of protons near the surface of the electrocatalyst is crucial to achieving a desired selectivity of NO_3_RR and a high production rate of ammonia; this factor is especially important for NO_3_RR commonly operated in unbuffered neutral electrolytes.^5,7,9,10^ Most published studies in the field of NO_3_RR focused on the design of better electrocatalysts, while reports on the modulation of local environments near the electrocatalyst, especially regarding the supply of protons, are relatively rare. One pioneering example was published in 2022 by Barile et al., demonstrating that the Nafion membrane coated on top of the Cu-based electrocatalyst could significantly improve the selectivity of NO_3_RR toward producing ammonia.^14^ The Nafion coating with enriched negatively charged sulfonate groups and mobile protons could serve as a proton supply to the adjacent electrocatalyst, which is beneficial for converting nitrate to ammonia. Recent studies also showed that the presence of Nafion coating,^15^ proton-rich ionic liquid,^16^ and ligands with terminal carboxylic acid^17^ near the electrocatalyst could facilitate the production of ammonia from nitrate. But all of these coatings, including Nafion and ionic liquid, are not porous, which should also retard the mass transfer of both nitrate and ammonia near the electrode surface and thus reduce the overall reaction rate. We thus reasoned that a highly porous membrane with the similar proton-rich characteristic of Nafion should be much more advantageous for boosting the NO_3_RR performance of the neighboring electrocatalyst.

Metal–organic frameworks (MOFs)^18,19^ are emerging porous materials with several appealing features including interconnected porosity, ultrahigh specific surface area, and highly tunable chemical functionality within the pore.^20−25^ Owing to these characteristics, researchers have applied MOFs and MOF-based materials for a range of catalytic applications.^26−31^ Although the poor chemical stability of most MOFs limits their direct use in aqueous environments,^32,33^ the rise of highly robust group(IV) metal-based MOFs,^33,34^ such as zirconium-based MOFs (Zr-MOFs), has opened up opportunities in employing MOFs in various aqueous electrochemical systems while preserving their crystalline and porous nature.^35−39^ Thus, a few very recent studies have utilized Zr-MOFs as electrocatalysts or porous supports of electrocatalytic species in NO_3_RR.^10,40,41^

However, it should be noted that most Zr-MOFs are intrinsically insulating for electrons.^42^ Therefore, charge-transport pathways such as the redox-hopping process are required to render catalytic sites within the Zr-MOF-based electrocatalyst electrochemically addressable.^42−45^ But such charge-hopping processes in Zr-MOFs are usually sluggish in aqueous electrolytes, which limits the resulting electrocatalytic performance.^42,46,47^ Thus, rather than employing the Zr-MOF as the porous support of electrocatalytic species, it is more advantageous to serve the Zr-MOF as a porous coating on top of another underlying electrocatalyst to adjust the local environment near the electrode surface and thus alter the reaction rates and selectivity. Zr-MOF here plays a similar role compared to membranes of Nafion and the ionic liquid mentioned previously, with a much higher porosity and tunability in chemical functionality. With this strategy, the electronic conduction within the porous MOF is no longer required, and the selection, design, and synthesis of the actual electrocatalyst and the functional MOF coating can be fully decoupled.^48^ Such concepts have been employed in electrochemical carbon dioxide reduction and alcohol oxidation in very recent studies reported by Hod and co-workers.^49−51^ In our recent work, we also found that the Zr-MOF with immobilized negatively charged sulfonate groups in its pores, SO_3_-MOF-808, can be used as an ion-gating coating on the electrocatalyst to preconcentrate the cationic analyte and repulse anionic interferents during the electrochemical sensing process.^52^ However, such concepts of utilizing a “catalytically inactive” Zr-MOF coating to further enhance the underlying electrocatalyst have not been reported for the NO_3_RR.

**Figure 1:**
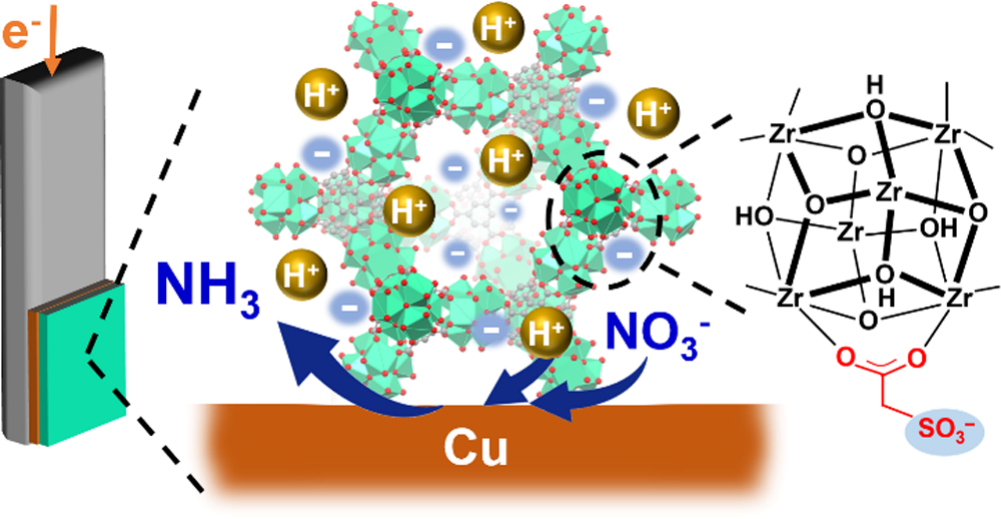
Schematic representation for the structure of SO_3_-MOF-808 and its role in supplying protons to the underlying Cu-based electrocatalyst to boost the selectivity of NO_3_RR.

In this study, one of the promising electrocatalysts for the NO_3_RR to produce ammonia, electrodeposited Cu, was first prepared on the electrode surface. A thin film of SO_3_-MOF-808 was thereafter coated on the surface of Cu to fabricate the bilayered modified electrode. With negatively charged sulfonate groups in its entire porous structure, the SO_3_-MOF-808 can serve as the “proton reservoir” during the NO_3_RR occurring on the underlying Cu, leading to enhanced selectivity toward the production of ammonia (see Figure 1). In contrast, the use of a MOF coating with positively charged trimethylammonium (TMA) groups significantly shifts the selectivity of NO_3_RR toward the formation of nitrite. The Cu/SO_3_-MOF-808 electrode can achieve a higher Faradaic efficiency for ammonia production, a higher nitrate-to-ammonia selectivity as well and a higher ammonia production rate compared to both the Cu electrode and that with the optimized Nafion coating, suggesting the role of SO_3_-MOF-808 as a more advantaged alternative to the commercially available Nafion for NO_3_RR.

## 2 Experimental Section

### 2.1 Chemicals

The purity and supplier of each chemical used for synthesizing MOF-808 and SO_3_-MOF-808 can be found in our recent work.^52^ Betaine hydrochloride (Sigma-Aldrich, ≥99%), ethanol (ECHO Chemical Co., Ltd., Taiwan, 95%), acetone (ECHO Chemical Co., Ltd., Taiwan, 98%), sulfuric acid (H_2_SO_4_, Honeywell Fluka, 95.0–98.0%), copper(II) sulfate pentahydrate (CuSO_4_, Duksan Pure Chemicals, 99%), Nafion 117 solution (Sigma-Aldrich, 5% in lower aliphatic alcohols and water), sodium sulfate (Na_2_SO_4_, Sigma-Aldrich, ≥99.0%), sodium nitrate (NaNO_3_, Thermo Scientific Chemicals, ≥98%), sodium nitrate-^15^N (Na^15^NO_3_, Alfa Aesar, ≥98%, ^15^N, ≥99%), ammonia sulfate ((NH_4_)_2_SO_4_, Duksan Pure Chemicals, 99.0%), sodium nitrite (NaNO_2_, Alfa Aesar, 98%), sulfuric acid-d_2_ (D_2_SO_4_, Sigma-Aldrich, 96–98 wt % in D_2_O, 99.5 atom % D), dimethyl sulfoxide-d_6_ (DMSO-*d*_6_, Sigma-Aldrich, 99.9 atom % D), hydrogen peroxide (H_2_O_2_, Honeywell Fluka, 30–31%), and nitric acid (HNO_3_, Honeywell Fluka, ≥65%) were purchased and used as received. Other chemicals used for quantifying the amounts of nitrite and ammonium ions in each electrolyte are the same as those used in our recent work.^53^ Deionized water was used for preparing all of the aqueous solutions.

### 2.2 Synthesis of MOFs

Synthetic procedures of activated MOF-808 powder and SO_3_-MOF-808 can be found in our recent work.^52^ To compare with the SO_3_-MOF-808 containing negatively charged sulfonate groups immobilized within the MOF pore, MOF-808 with immobilized positively charged TMA groups was also synthesized by performing the solvent-assisted ligand incorporation (SALI).^54,55^ The procedure is described as follows. Betaine hydrochloride (81.2 mg, 18 equiv to the nodes of MOF-808) was dissolved in 19 mL of ethanol, and 60 mg of the activated MOF-808 powder was dispersed in the resulting solution. The mixture was sealed in a glass vial and placed in an oven at 60 °C for 24 h. The obtained solid was washed with 20 mL of ethanol three times through centrifugation over the course of overnight and was thereafter subjected to vacuum activation at 60 °C overnight. The obtained powder was designated as “TMA-MOF-808.”

### 2.3 Preparation of Electrodes

Carbon paper electrodes (3 cm × 1 cm, CeTech Co., Ltd., Taiwan, 0.31 mm thick, through plane resistance <5 mΩ cm^2^) were washed with ethanol and acetone by following the previously reported procedure.^39^ An insulating polyamide tape was used to obtain an exposed geometric area of 1 cm^2^ (1 cm × 1 cm) on the carbon paper, and the obtained electrode was further cleaned by a UV-ozone cleaner (Jelight Company, Inc., Model No. 42) for 15 min.^39^ Thereafter, the prepared carbon paper electrode was subjected to the electrodeposition of metallic copper by following the method reported previously.^56^ An aqueous solution containing 0.05 M H_2_SO_4_ and 0.02 M CuSO_4_ was employed as the electrolyte for electrodeposition, and the cleaned carbon paper, a platinum foil, and Ag/AgCl/NaCl (3 M) (BASI) were utilized as the working electrode (WE), counter electrode (CE), and reference electrode (RE), respectively. A constant potential of −1.655 V vs. Ag/AgCl/NaCl (3 M) was applied to the WE for 100 s. It is worth noting that for preparing the Cu-based electrocatalyst for NO_3_RR in the previous work, 400 s was used for such electrodeposition on foam-type electrodes.^56^ However, the use of 400 s on carbon-paper electrodes here resulted in thin-film detachment during rinsing; 100 s of deposition was thus selected after optimizing the electrodepositing process. The obtained electrode was immediately removed from the electrolyte and rinsed with water. After drying it in a vacuum oven at 80 °C overnight, the electrode with “Cu” electrocatalyst was obtained.

To further deposit the MOF coating on top of the Cu electrode, the drop-casting process reported in our previous work was employed.^52^ Accurately weighed 12 mg of MOF-808, SO_3_-MOF-808, or TMA-MOF-808 powder was dispersed in 1 mL of acetone by ultrasonication for 10 min, and 24 μL of the resulting suspension was drop-cast on the surface of the Cu electrode. The obtained electrodes after drying in an oven at 60 °C were designated as “Cu/MOF-808,” “Cu/SO_3_-MOF-808,” and “Cu/TMA-MOF-808,” respectively. The loading of the MOF on the electrode surface is 0.288 mg/cm^2^. To optimize the MOF loading, the drop-casting process was performed twice and three times for SO_3_-MOF-808 to achieve loadings of 0.576 and 0.864 mg/cm^2^, respectively, and the suspension with a concentration of 6 mg/mL was used for the drop-casting process to attain a loading of 0.144 mg/cm^2^. SO_3_-MOF-808 with a loading of 0.288 mg/cm^2^, which is the optimal loading as discussed later, was also coated on the cleaned carbon paper without any Cu; the obtained electrode was named “SO_3_-MOF-808.”

For comparison, the commercially available Nafion coating was also deposited on top of the Cu electrode. 0.1 mL of the Nafion 117 solution (5%) was first mixed with 4.9 mL of ethanol, and 24 μL of the resulting solution was drop-cast on the surface of the Cu electrode. After drying at 60 °C, the obtained electrode was designated as “Cu/Nafion,” with a Nafion loading of approximately 0.024 mg/cm^2^. By the adjustment of the concentration of the diluted Nafion solution for drop-casting, Cu electrodes with the Nafion loadings of 0.012, 0.048, 0.120, and 0.240 mg/cm^2^ were also fabricated.

### 2.4 Electrochemical Experiments

Three-electrode setup with the carbon-paper-based electrode, Pt foil, and Ag/AgCl/NaCl (3 M) as the WE, CE, and RE was used for all electrochemical experiments. A CHI1205C electrochemical workstation (CH Instruments, USA) was employed for all electrochemical tests. In order to reduce all surface-oxidized copper back to its metallic state, a constant potential of −0.79 V vs. SHE was applied to the WE for 10 min in the aqueous electrolyte containing 1.0 M of Na_2_SO_4_ prior to every electrolytic, linear sweep voltammetry (LSV), or cyclic voltammetric (CV) experiment; this procedure follows the protocol reported previously.^53^ All electrochemical tests were performed with the use of a two-compartment electrochemical cell. For all electrolytic experiments, 15 mL of the aqueous solution containing 0.5 M of Na_2_SO_4_ and a certain concentration of NaNO_3_ (0.5 M, 100, 50, 20, or 10 mM) was added in both compartments. Electrolytes in both compartments were bubbled with Ar gas for 20 min before each electrolysis. The electrolyte in the compartment with WE and RE was collected after electrolysis for product analysis.

Concentrations of nitrite ions and ammonium ions in each electrolyte after the electrolysis were determined by utilizing UV–visible spectroscopy.^53^ For quantifying ammonium ions, reagents with sodium hydroxide, sodium citrate, salicylic acid, sodium hypochlorite, and sodium pentacyanonitrosylferrate were used and (NH_4_)_2_SO_4_ was employed as the standard. For quantifying nitrite ions, the reagent with sulfanilamide, N-(1-Naphthyl)ethylenediamine dihydrochloride, and phosphoric acid was used with NaNO_2_ as the standard. Detailed protocols for product analysis can be found in Section S1 of the Supporting Information.

For electrolytic experiments with the ^15^N isotope, the Cu/SO_3_-MOF-808 electrode was subjected to electrolysis at −1.19 V vs. SHE for 1 h in an aqueous electrolyte containing 0.5 M Na_2_SO_4_ and 0.5 M of Na^15^NO_3_. The pH of the collected electrolyte was then adjusted to 2.0 prior to ^1^H nuclear magnetic resonance (NMR) measurements; see the detailed protocol in our previous work.^53^

### 2.5 Instruments

A UV-2600 spectrometer (Shimadzu) was used for product analysis. Grazing incidence X-ray diffraction (GIXRD) patterns of modified electrodes and powder X-ray diffraction (PXRD) data of MOFs were collected using a SmartLab (Rigaku). Details regarding the instruments and sample preparations for scanning electron microscopy (SEM), nitrogen gas adsorption–desorption measurements, Fourier transform infrared (FTIR) measurements, and NMR measurements are the same as those reported in our recent work.^52^ To quantify the loading of Cu, the Cu electrode (1 cm^2^) was immersed in 0.75 mL of H_2_SO_4_ and 0.25 mL of H_2_O_2_ in a microwave vial, and the crimped vial was subjected to microwave digestion by using an Initiator+ (Biotage) at 150 °C for 20 min. The resulting mixture was diluted to 40 mL by adding 3% HNO_3_ aqueous solution, and the filtration was performed before the analysis with inductively coupled plasma optical emission spectrometry (ICP-OES, JY 2000-2, Horiba Scientific).

## 3 Results and Discussion

### 3.1 Characterizations of MOFs

**Figure 2:**
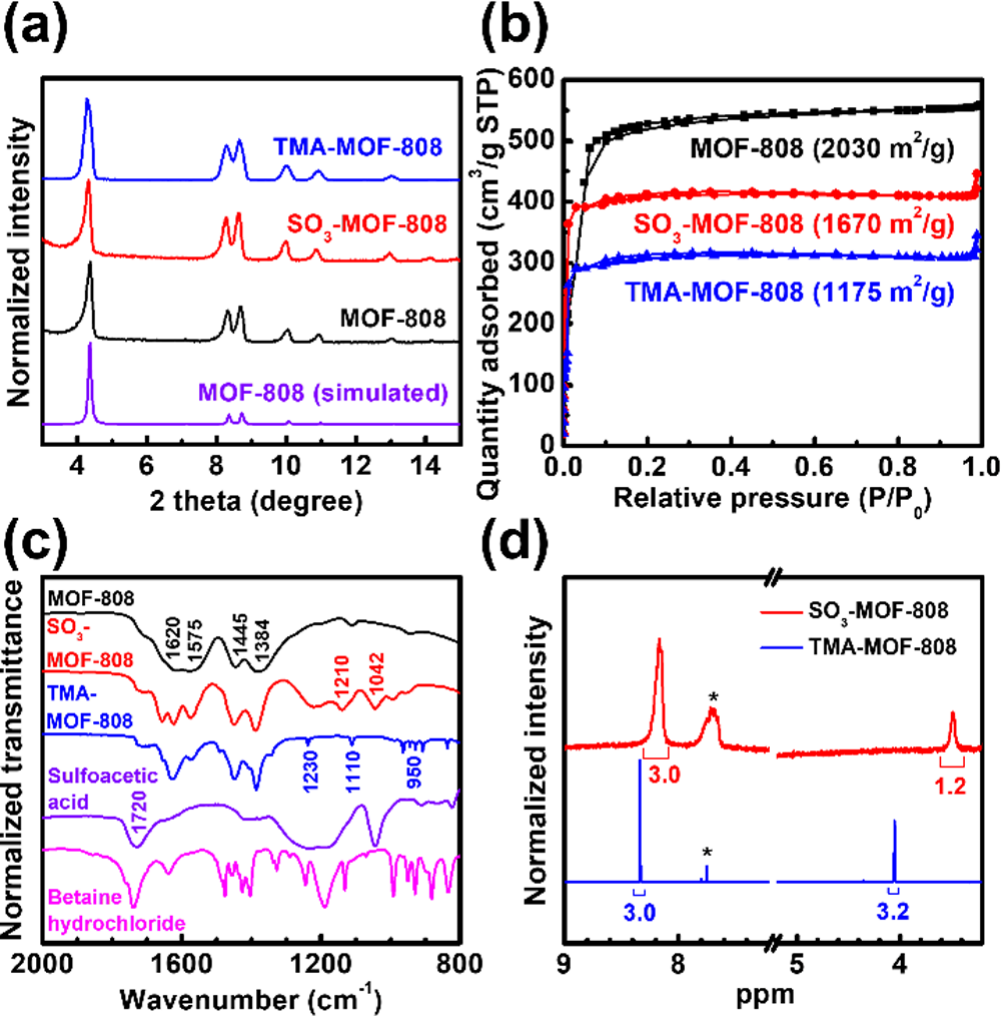
(a) PXRD patterns, (b) N_2_ adsorption–desorption isotherms, and (c) FTIR spectra of the MOFs. Simulated pattern of MOF-808 is shown in (a) and BET surface areas are listed in (b). FTIR spectra of sulfoacetic acid and betaine hydrochloride are also shown in (c). (d) NMR spectra of digested MOFs; peaks of the residual formate modulator are marked with stars.

Powder of MOF-808 was first synthesized, and as shown in Figures 2a and S1a, it is composed of octahedral microcrystals with its PXRD pattern consistent with the simulated one. Nitrogen adsorption–desorption isotherm of MOF-808 (Figure 2b) reveals a feature of microporous materials with a Brunauer–Emmett–Teller (BET) surface area of 2030 m^2^/g, which agrees well with that of MOF-808 reported previously;^52,57^ it indicates that the synthesis of phase-pure MOF-808 was successful. SALI was then utilized to coordinate the sulfoacetic acid onto the nodes of MOF-808 by following the synthetic procedure reported in our recent work.^52^ On the other hand, MOF-808 functionalized with positively charged TMA groups was also synthesized via employing SALI with the betaine hydrochloride. As shown in Figure 2a,b, the crystallinity of MOF-808 can be preserved after the installation of both ligands and the major porosity of the framework is still present in both SO_3_-MOF-808 and TMA-MOF-808. SEM images collected at both high and low magnifications also reveal that there is no morphological change after the incorporation of each ligand (Figure S1). Density functional theory (DFT) pore size distributions were then extracted from the isotherms shown in Figure 2b, and the results are plotted in Figure S2. MOF-808 has a major pore size centered at 1.7 nm, in agreement with the pore present in its crystalline structure. This pore size decreases to 1.5 and 1.1 nm after the installation of sulfoacetate and betaine, respectively, which further indicates the successful incorporation of both ligands within the entire MOF structure. FTIR spectra of MOF-808, SO_3_-MOF-808, and TMA-MOF-808 are shown in Figure 2c. For comparison, the spectra of both ligands are also presented. Characteristic peaks of MOF-808, including those for C=O stretching vibration (1620 cm^–1^), C–O–C asymmetric vibration (1575 cm^–1^), C=C vibration of the aromatic ring (1445 cm^–1^), and O–C–O symmetric vibration (1384 cm^–1^),^52^ can be observed in spectra of all the three MOFs. In addition, peaks for the S=O stretching from sulfonate groups located at 1210 and 1042 cm^–1^ can be observed in the spectrum of SO_3_-MOF-808,^52^ and characteristic peaks of betaine at 1230, 1110, and around 950 cm^–1^ are present in the spectrum of TMA-MOF-808. Furthermore, the strong peak located at 1720 cm^–1^ in the FTIR spectra of both ligands, corresponding to the C=O bond of the free carboxylic acid, is also less obvious in the spectra of both functionalized MOFs. Agreeing with our previous findings for SO_3_-MOF-808,^52^ results here indicate the successful coordination of both ligands on hexa-zirconium nodes of MOF-808 mainly through carboxylate groups. Both SO_3_-MOF-808 and TMA-MOF-808 were then digested for NMR measurements in order to quantify the loading of ligands. As revealed in Figure 2d, the peak from three protons of the trimistic acid, the linker of MOF-808, can be found at around 8.3 ppm in the spectra of both digested MOFs. Furthermore, peaks at 4.1 and 3.5 ppm correspond to two protons from the betaine and two protons from the sulfoacetic acid, respectively. From the integrated areas listed in Figure 2d and the linker-to-node ratio of two in MOF-808, loadings of the coordinated sulfoacetate in SO_3_-MOF-808 and coordinated betaine in TMA-MOF-808 were determined as 1.2 and 3.2 ligands per node, respectively. All the above findings indicate the successful immobilization of negatively charged sulfonate-based ligands and positively charged TMA-based ligands in MOF-808, respectively, without damaging the crystallinity and clogging the major porosity of the Zr-MOF.

### 3.2 NO_3_RR under a High Concentration of Nitrate

**Figure 3:**
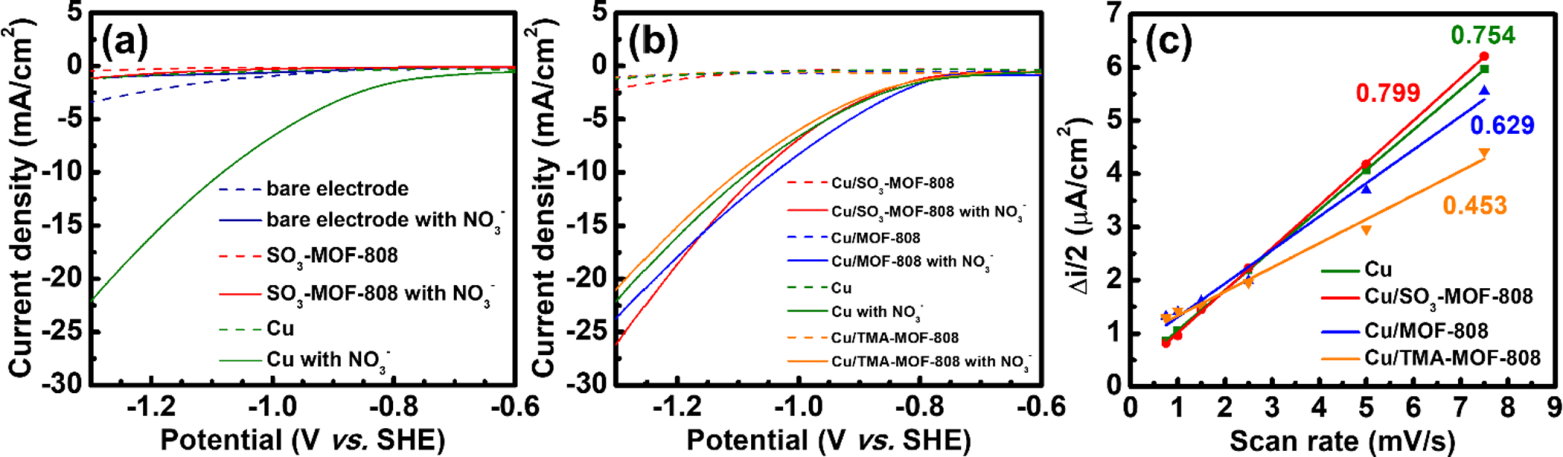
(a,b) LSV curves of various electrodes, measured in the aqueous electrolyte containing 1.0 M Na_2_SO_4_ or that containing 0.5 M Na_2_SO_4_ and 0.5 M NaNO_3_ (marked as “with NO_3_^–^”). Scan rate: 10 mV/s. (c) Plot of the average non-Faradaic current (Δ*i*/2) versus scan rate obtained from CV data shown in Figure S3. Values of slopes of linear fitting curves are listed in (c).

To fabricate modified electrodes that are electrocatalytically active for the NO_3_RR, metallic Cu was electrodeposited on carbon-paper electrodes. ICP-OES measurements of the digested Cu electrode reveal an average Cu loading of 0.252 mg/cm^2^. MOF-808, SO_3_-MOF-808, or TMA-MOF-808 were then further coated on top of the copper-modified electrode to prepare Cu/MOF bilayered electrodes. LSV analysis was thereafter performed to preliminarily probe the overall electrocatalytic activity of these electrodes for the NO_3_RR in aqueous electrolytes containing 1.0 M Na_2_SO_4_ and 0.5 M Na_2_SO_4_/0.5 M NaNO_3_, respectively. It is worth mentioning that a high concentration of nitrate, i.e., 500 mM, was first used to render a sufficient supply of nitrate ions to the electrode surface;^11,53,58^ electrocatalysis at lower concentrations of nitrate will be discussed later in Section 3.3. As revealed in Figure 3a, both the bare carbon-paper electrode and SO_3_-MOF-808-modified carbon paper show negligible catalytic current responses after adding nitrate compared to the Cu-modified carbon paper, clearly suggesting that copper is the only active electrocatalyst for NO_3_RR. LSV curves of all Cu-based modified electrodes were then tested. As shown in Figure 3b, all four electrodes achieve similar catalytic current responses in the electrolyte containing 0.5 M of NaNO_3_. This result indicates that the overall reaction rate, which should include NO_3_RR to ammonia, NO_3_RR to nitrite, and HER for copper-based electrocatalysts,^5,11,12^ does not show obvious differences in the presence of various Zr-MOF coatings. Electrochemically active surface areas (ECSA) of the four Cu-based modified electrodes were then gauged by measuring their non-Faradaic responses in the CV curves. CV curves of electrodes measured in the electrolyte containing 0.5 M of Na_2_SO_4_ and 0.5 M of NaNO_3_ within the potential range that does not initiate Faradaic reactions are shown in Figure S3, and values of non-Faradaic current averaged from the anodic and cathodic sides of CV curves (Δ*i*/2) are plotted with the scan rate in Figure 3c; the slope in the plot is proportional to the ECSA of the electrode (see detailed discussions in the Supporting Information).^59^ It can be observed that except for the Cu/TMA-MOF-808 which possesses a slightly reduced ECSA, all other modified electrodes have similar ECSA. Since all Zr-MOF coatings are porous while fully insulating for electrons, such a MOF coating should not contribute to any ECSA of the electrode, and the ECSA should solely come from the underlying copper and carbon paper; this finding is consistent with those in previous studies reporting such redox-innocent Zr-MOF coatings.^49,52^ Results in Figure 3 indicate that both the overall reaction rate and ECSA of the underlying Cu catalyst are barely affected by the Zr-MOF coating used here; electrolysis and further product analysis are required to examine the effect of the MOF coatings on the reaction rates and selectivity of the NO_3_RR.

Electrolytic experiments in electrolytes containing 0.5 M Na_2_SO_4_ and 0.5 M NaNO_3_ were then conducted for 1 h at various applied potentials. Since both HER and NO_3_RR consume protons near the electrode surface, the increase in the pH value may damage the structure of Zr-MOF at high reaction rates; it is thus crucial to first examine the structural integrity of the MOF coating during the electrolysis. As revealed in Figure S4 and corresponding discussions in the Supporting Information, the SO_3_-MOF-808 coating can preserve its crystallinity after electrolytic experiments at −0.99, −1.09, and −1.19 V vs. SHE, but the degradation of the MOF occurred when −1.29 V was applied. In addition, NMR data also suggest the negligible leaching of coordinated sulfonate-based ligands from the MOF coating during the electrolysis at −1.19 V vs. SHE. Thus, to utilize the MOF coating while preserving its crystalline and porous structure, all following discussions will focus on electrolytic experiments at −0.99, −1.09, and −1.19 V.

**Figure 4:**
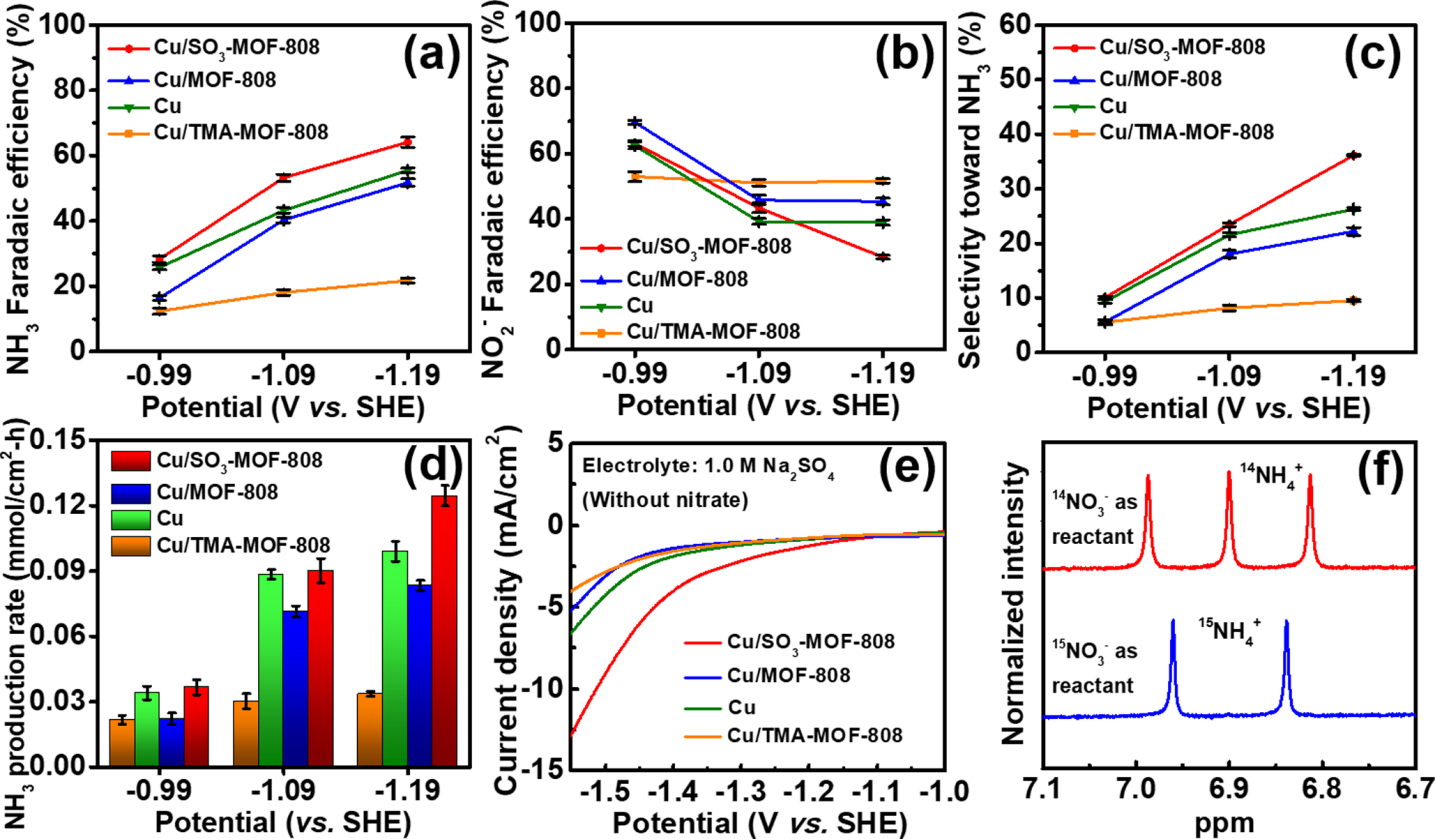
(a) FE for NO_3_RR to ammonia, (b) FE for NO_3_RR to nitrite, (c) selectivity of NO_3_RR toward ammonia, and (d) ammonia production rates of various Cu-based modified electrodes, obtained from electrolytic experiments conducted at various applied potentials for 1 h in aqueous electrolytes containing 0.5 M of Na_2_SO_4_ and 0.5 M of NaNO_3_. Error bars in (a–d) indicate one standard deviation away from the averaged results, obtained from three separate electrolytic experiments. (e) LSV curves of various Cu-based modified electrodes measured in 1.0 M of Na_2_SO_4_ without nitrate ions, at a scan rate of 10 mV/s. (f) NMR spectra of electrolytes after electrolytic experiments with Cu/SO_3_-MOF-808 electrodes at −1.19 V vs. SHE for 1 h by serving 0.5 M Na^15^NO_3_ and 0.5 M Na^14^NO_3_ as reactants, respectively.

Chronoamperometric data of all Cu-based modified electrodes recorded during electrolytic experiments at −0.99, −1.09, and −1.19 V are plotted in Figure S5, and the electrolyte after each electrolysis was subjected to the product analysis to quantify its concentrations of nitrite and ammonium ions; see calibration curves and UV–visible data in Figures S6–S8 and detailed protocols in Section S1 of the Supporting Information. Faradaic efficiencies (FE) for NO_3_RR to ammonia, FE for NO_3_RR to nitrite, and the selectivity of NO_3_RR toward ammonia were then calculated (see calculating details in Section S6 of the Supporting Information), and the results are plotted in Figure 4a–c; also, see values listed in Table S1. In the presence of 0.5 M nitrate, the bare Cu electrocatalyst can achieve more than 80% of overall FE for NO_3_RR with suppressed HER at all applied potentials. However, the selectivity of the NO_3_RR is, in general, more favorable toward the production of nitrite at such a high concentration of reactant, and the selectivity toward ammonia increases with the increasing overpotential. With the MOF-808 coating on top of the Cu electrocatalyst, the resulting modified electrode shows similar selectivity compared to the bare Cu electrode, with slightly lower values of selectivity toward ammonia. On the other hand, the negatively charged SO_3_-MOF-808 coating can increase both the FE and selectivity of the underlying Cu electrocatalyst toward the production of ammonia and this enhancement is most significant at the applied potential of −1.19 V. We hypothesized that the negatively charged sulfonate groups in the MOF should enrich more protons near the surface of the underlying copper, facilitating the NO_3_RR to generate ammonia rather than that to produce nitrite. To examine the role of negatively charged SO_3_-MOF-808 as the proton reservoir, positively charged TMA-MOF-808 coating was also employed for comparison. As shown in Figure 4, with the TMA-MOF-808 coating, the selectivity of NO_3_RR occurring on copper becomes much more favorable toward nitrite, with a selectivity of more than 90% toward nitrite at every applied potential. This finding indicates that the positively charged TMA groups in the coating can play a role in repulsing protons from the copper surface, which is more beneficial for the production of nitrite that only requires two protons compared to the nine-proton reaction for generating ammonia. Production rates of ammonia at various applied potentials are plotted in Figure 4d. At −1.19 V, the Cu/SO_3_-MOF-808 electrode can achieve a production rate of 0.125 mmol/cm^2^ h, which is higher than that achieved by the pristine Cu electrode (0.099 mmol/cm^2^ h). For comparison, electrolytic experiments and product analysis were also performed with bare carbon paper electrodes and SO_3_-MOF-808-modified carbon papers without Cu. As revealed in Figure S9, although the SO_3_-MOF-808 coating can slightly increase the overall FE for the NO_3_RR, both electrodes show minor FE for the NO_3_RR and negligible production rates of ammonia compared to those achieved by the copper-modified electrode, again suggesting that copper is the active electrocatalyst for the NO_3_RR here. It is worth mentioning that the loading of the SO_3_-MOF-808 coating used here, i.e., 0.288 mg/cm^2^, has been optimized as well (see Figures S10 and S11 and corresponding discussions in the Supporting Information).

To further verify the proton-enriching and proton-repulsing effects provided by MOF coatings, LSV data of all Cu-based modified electrodes were collected in the absence of nitrate ions to investigate their reaction rates of HER, the only reaction consuming protons here. It should be noted that the promotion of HER by a proton-supplying Zr-MOF coating has been reported by a previous study.^35^ As revealed in Figure 4e, compared to the pristine Cu electrode, the Cu/SO_3_-MOF-808 electrode with negatively charged sulfonate groups near the surface can largely facilitate HER, while the Cu/TMA-MOF-808 electrode with positively charged TMA groups can obviously suppress HER. Results here again suggest the role of the SO_3_-MOF-808 coating to concentrate more protons near the electrode surface and thus enhance the selectivity of NO_3_RR toward ammonia in the presence of nitrate ions. Electrolysis was also performed with the ^15^N isotope, and as shown in Figure 4f, only signals from ^15^NH_4_^+^ can be found when the Na^15^NO_3_ was served as the reactant.

### 3.3 NO_3_RR in Low Concentrations of Nitrate

**Figure 5:**
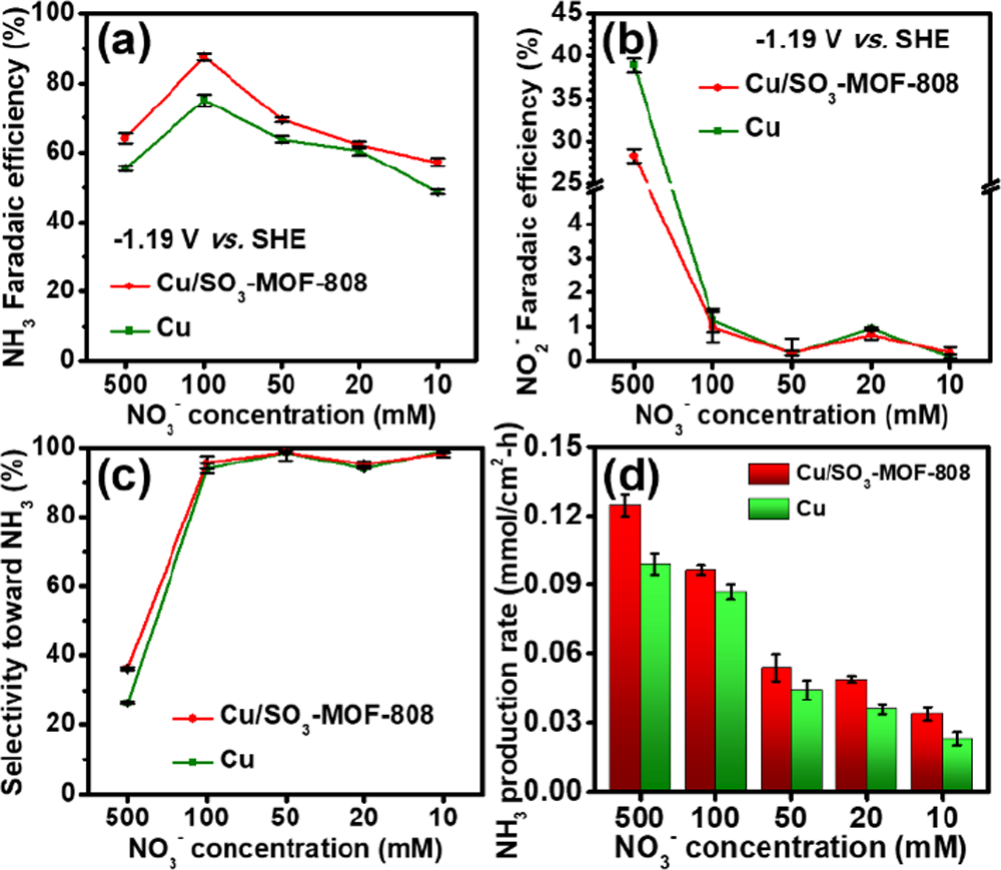
(a) FE for NO_3_RR to ammonia, (b) FE for NO_3_RR to nitrite, (c) selectivity of NO_3_RR toward ammonia, and (d) ammonia production rates of Cu/SO_3_-MOF-808 and Cu electrodes, obtained from electrolytic experiments performed at −1.19 V vs. SHE for 1 h in aqueous electrolytes containing 0.5 M Na_2_SO_4_ and various concentrations of NaNO_3_. Error bars indicate one standard deviation away from the averaged results, obtained from three separate electrolytic experiments.

Although the SO_3_-MOF-808 coating can enrich protons near the copper surface and enhance the selectivity of the NO_3_RR toward ammonia, it may also repulse nitrate ions from the surface; this phenomenon may become less beneficial for the NO_3_RR especially when the concentration of nitrate ions in the electrolyte is low. Thus, to verify the applicability of such MOF coatings in NO_3_RR, especially for the practical application with the wastewater containing a low concentration of nitrate, electrolytic experiments were further conducted in electrolytes containing 100, 50, 20, and 10 mM nitrate at the optimal applied potential of −1.19 V vs. SHE. Cu/SO_3_-MOF-808 and pristine Cu electrodes were employed. Chronoamperometric and UV–visible data are shown in Figures S12–S14, and results are plotted in Figure 5 (also see values listed in Table S2). From Figure 5b,c, it can be clearly seen that the production of nitrite is significantly suppressed when the concentration of the reactant is equal to or lower than 100 mM; the low concentration of nitrate ions allows their complete conversion into ammonia by the copper electrocatalyst. However, as shown in Figure 5a, the FE for ammonia drops with the decreasing concentration of nitrate when the concentration is lower than 100 mM since the insufficient supply of nitrate ions also promotes the FE for HER. On the other hand, the production rate of ammonia decreases with decreasing concentration of nitrate, as revealed in Figure 5d. At the optimal concentration of nitrate, i.e., 100 mM, a FE of 87.5% and a production rate of 0.097 mmol/cm^2^ h for producing ammonia can be achieved by Cu/SO_3_-MOF-808, which are much better than those achieved by the pristine Cu electrode (75.0% and 0.087 mmol/cm^2^ h). In addition, data in Figure 5 also suggest that the Cu/SO_3_-MOF-808 electrode can exhibit both a higher FE and a higher production rate for ammonia than the Cu electrode in every concentration of nitrate ions down to 10 mM. Findings here indicate the generalizability of utilizing such an SO_3_-MOF-808 coating on top of the Cu electrocatalyst in enhancing the NO_3_RR performance, even in the electrolyte containing a low concentration of nitrate.

### 3.4 Comparison to Nafion Coatings and Performances Reported in the Literature

**Figure 6:**
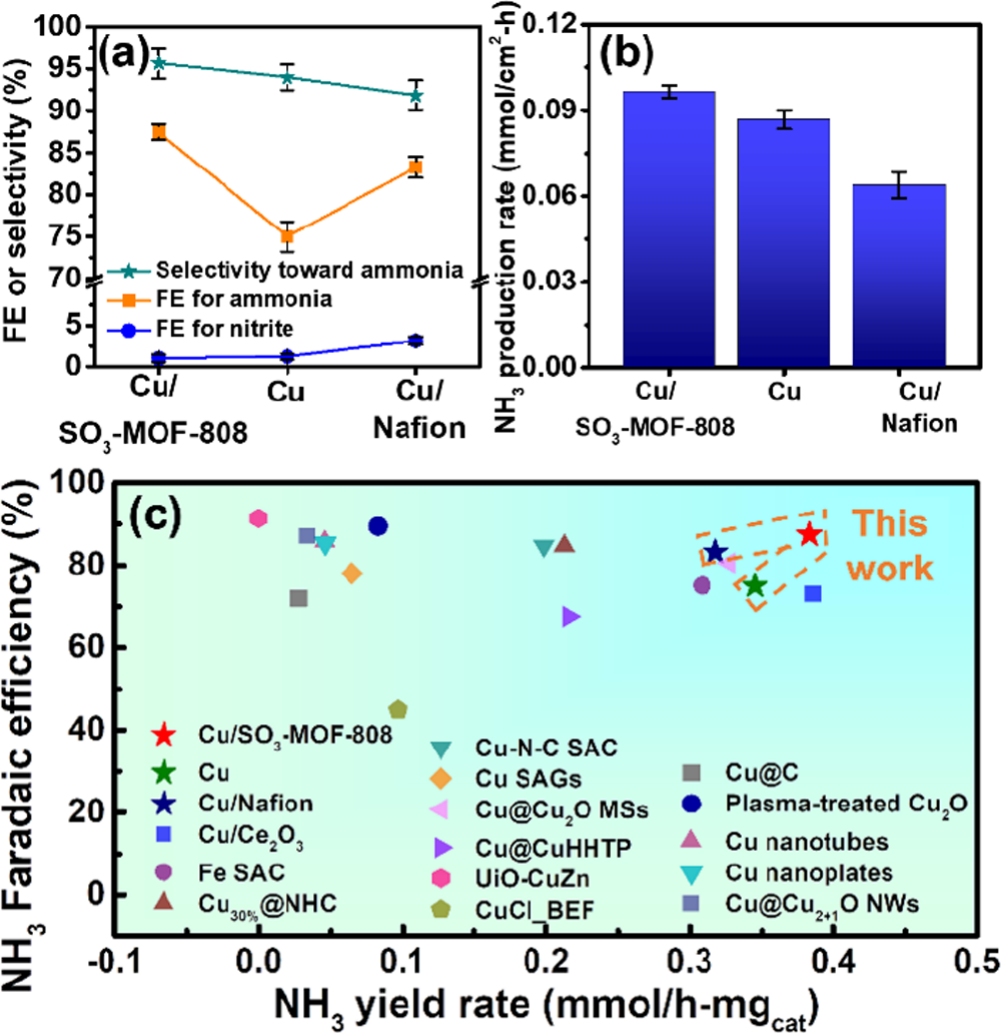
(a) FE for NO_3_RR to ammonia, FE for NO_3_RR to nitrite, and selectivity of NO_3_RR toward ammonia, and (b) ammonia production rates of Cu/SO_3_-MOF-808, Cu/Nafion, and Cu electrodes, obtained from electrolytic experiments performed at −1.19 V vs. SHE for 1 h in aqueous electrolytes containing 0.5 M Na_2_SO_4_ and 100 mM of NaNO_3_. Error bars in parts a and b indicate one standard deviation away from the averaged results, obtained from three separate electrolytic experiments. (c) Comparison to reported electrocatalysts for NO_3_RR, obtained from the data in Table S4.

Nafion has been reported as the coating on top of the electrocatalyst to boost the resulting performance of NO_3_RR.^14,15^ Thus, the electrode with the SO_3_-MOF-808 coating was compared with that with the commercially available Nafion coating under optimal operating conditions, i.e., 100 mM nitrate and the applied potential of −1.19 V vs. SHE. The loading of the Nafion coating on top of the Cu electrode was first optimized, and the corresponding electrolytic data are shown in Figures S15 and S16. It can be observed that with a small loading of Nafion on Cu, the overall FE for the NO_3_RR is even reduced, presumably owing to the less uniform coverage of the Nafion coating. Both the FE and production rate for ammonia reached maxima with a Nafion loading of 0.024 mg/cm^2^. With higher loadings of Nafion on the Cu surface, the thick and less porous Nafion coating may start to repulse nitrate ions from the electrode surface, resulting in decreased FE and production rates for ammonia and the promoted FE for HER. Thus, Nafion coating with a loading of 0.024 mg/cm^2^ was selected for further comparison (also see results listed in Table S3). As revealed in Figure 6a,b, the Cu/Nafion electrode can achieve a much better FE for ammonia (83.3%) compared to that achieved by the Cu electrode (75.0%), but both the selectivity of the NO_3_RR toward ammonia and the production rate of ammonia were slightly reduced in the presence of the Nafion coating. On the other hand, the electrode with the SO_3_-MOF-808 coating can outperform both the pristine Cu electrode and Cu/Nafion electrode in the FE for ammonia, selectivity of NO_3_RR toward ammonia, and production rate of ammonia. Although the SO_3_-MOF-808 is not electrochemically active, its interconnected porosity and relatively large pore sizes (ca., 1.8 nm) should render a faster mass transfer of both nitrate ions and protons from the electrolyte to the Cu surface compared to that in nonporous Nafion, leading to its better FE for ammonia and a much faster ammonia production rate. Findings here clearly suggest that SO_3_-MOF-808 can act as a better coating material to concentrate protons near the surface of the electrocatalyst than commercially available Nafion.

According to the mass loading of the Cu electrocatalyst deposited on the electrode determined by ICP-OES (0.252 mg/cm^2^), the production rates of ammonia shown in Figure 6b were converted into the NH_3_ yield rate of electrocatalysts (in mmol/h mg_cat_). Results here were then compared with performances reported in recent studies in the field of NO_3_RR.^5,10−12,60−70^ It should be noticed that 15 examples from the literature with the use of various concentrations of nitrate ranging from 100 mM to 50 ppm as reactants were included for the comparison. The detailed comparison and the full information on all reported materials are listed in Table S4 in the Supporting Information, and FE for ammonia and ammonia yield rates are plotted in Figure 6c. From Table S4, it can be seen that those studies using high concentrations of nitrate (100 mM, 20 mM, or 500 ppm) could achieve comparable or lower NH_3_ yield rates compared to those reported in this work, but their FE for ammonia are all lower than that achieved by the Cu/SO_3_-MOF-808 here. On the other hand, published studies utilizing low concentrations of nitrate (200, 100, or 50 ppm) could achieve high FE for ammonia, but their NH_3_ yield rates are in general much lower than those achieved here. With the SO_3_-MOF-808 coating to modulate the environment near the surface of the electrocatalyst, the Cu electrocatalyst can achieve among the top performance in NO_3_RR compared to these published results, as revealed in Figure 6c.

## 4 Conclusions

A zirconium-based MOF with negatively charged sulfonate groups immobilized within the pore, SO_3_-MOF-808, can be synthesized by performing the postsynthetic modification with MOF-808. The MOF can be employed as a porous coating on top of the copper-based electrocatalyst aiming for the NO_3_RR in neutral aqueous solutions while preserving its crystallinity and sulfonate loading after the electrolysis at an applied potential of −1.19 V vs. SHE. Owing to its interconnected porosity and electrically insulating nature, the MOF coating barely alters both the ECSA and the overall reaction rate of the electrode. However, by means of the negatively charged sulfonate groups concentrating protons near the surface of the catalyst, both the FE for ammonia production as well as the nitrate-to-ammonia selectivity can be significantly increased. In contrast, the use of MOF coating with positively charged trimethylammonium groups to repulse protons strongly facilitates the conversion of nitrate to nitrite, with selectivity of more than 90% at all applied potentials in electrolytes containing 0.5 M of nitrate. From electrolytic experiments in electrolytes containing various concentrations of nitrate, it was found that 100 mM of the reactant can result in the optimal performance for producing ammonia. At the optimal operating condition, the Cu electrode with the SO_3_-MOF-808 coating can achieve a FE of 87.5% for ammonia, a nitrate-to-ammonia selectivity of 95.6%, and an ammonia production rate of 0.097 mmol/cm^2^ h (0.383 mmol/h mg_cat_), outperforming all of those achieved by the bare Cu electrode (75.0%; 93.9%; 0.087 mmol/cm^2^ h; 0.345 mmol/h mg_cat_). Nafion coating with an optimized loading was also compared, and the SO_3_-MOF-808 coating can even outperform it.

The findings here suggest that although the Zr-MOF is not electrically conductive nor electrochemically active, it can act as a porous coating on top of the electrocatalyst to modulate the proton concentration near the surface of the catalyst and thus adjust the reaction rates and selectivity; it can even outperform the commercially available Nafion in this role in the NO_3_RR. Ongoing work is focusing on utilizing such proton-supplying MOF coatings in other proton-coupled electrochemical processes.

## Supporting Information Available

Procedures for product analysis, SEM images and pore size distributions, CV analysis for gauging ECSA, chemical stability of MOF thin films after electrolysis, chronoamperometric and UV–visible data (0.5 M of nitrate), calculation of FE and selectivity, additional electrolytic data (0.5 M of nitrate), chronoamperometric and UV–visible data (various nitrate conc.), chronoamperometric and UV–visible data of Nafion-cast electrodes, comparison to performances in the literature (PDF)
