# Supplementary material for: Sulfonate-Functionalized Metal–Organic Framework as a Porous “Proton Reservoir” for Boosting Electrochemical Reduction of Nitrate to Ammonia
Source: ACS Appl Mater Interfaces. 2024 Nov 1;16(45):62185–94. doi: 10.1021/acsami.4c14786 (PMC11565520; doi:10.1021/acsami.4c14786)
Supplement: Supplementary file 1 — am4c14786_si_001.pdf [file am4c14786_si_001.pdf]

Supporting Information

Sulfonate-Functionalized Metal–Organic Framework as a Porous "Proton Reservoir" for Boosting Electrochemical Reduction of Nitrate to Ammonia

Yun-Shan Tsai,[a] Shang-Cheng Yang,[a] Tzu-Hsien Yang,[a,b] Chung-Huan Wu,[a] Tzu-Chi Lin,[a] and Chung-Wei Kung [a,b*]

[a] Department of Chemical Engineering, National Cheng Kung University, 1 University Road, Tainan City, Taiwan

[b] Program on Key Materials, Academy of Innovative Semiconductor and Sustainable Manufacturing, National Cheng Kung University, 1 University Road, Tainan City, Taiwan

* Corresponding author: cwkung@mail.ncku.edu.tw

Table of contents

Section	Page number
Table of contents	S2
S1. Procedures for product analysis	S3
S2. SEM images and pore size distributions	S4
S3. CV analysis for gauging ECSA	S5
S4. Chemical stability of MOF thin films after electrolysis	S6
S5. Chronoamperometric and UV-visible data (0.5 M of nitrate)	S8
S6. Calculation of Faradaic efficiencies (FE) and selectivity	S12
S7. Additional electrolytic data (0.5 M of nitrate)	S13
S8. Effect of the MOF loading on top of the Cu electrode	S14
S9. Chronoamperometric and UV-visible data (various nitrate conc.)	S16
S10. Chronoamperometric and UV-visible data of Nafion-cast electrodes	S19
S11. Comparison to performances in the literature	S21
S12. References	S23

S1. Procedures for product analysis

For quantifying the concentration of ammonium ions in the electrolyte collected after the electrolysis, the following analysis was performed. The electrolyte was first diluted to the detecting range by adding a certain amount of water, and 2 mL of the diluted electrolyte was mixed with 2 mL of sodium hydroxide aqueous solution (1.0 M) containing 5 wt% of sodium citrate and 5 wt% of salicylic acid. At the same time, 1 mL of sodium hypochlorite aqueous solution (0.05 M) was added into 0.2 mL of sodium pentacyanonitrosylferrate(Ⅲ) aqueous solution (1 wt%) to form another solution. Both solutions were then mixed, and the obtained solution was placed at room temperature for 2 h. UV-visible spectrum of the solution was then collected immediately, and the intensity of the absorption peak located at 654 nm was recorded. To obtain the calibration curve for quantification, 2 mL of $(NH_4)_2SO_4$ aqueous solution with a known concentration of ammonium was used to replace the diluted electrolyte.

For quantifying the concentration of nitrite ions in the electrolyte, the following experiments were conducted. 1.0 g of sulfanilamide, 0.05 g of N-(1-Naphthyl)ethylenediamine dihydrochloride, 2.5 mL of phosphoric acid, and 1.25 of water were mixed to obtain the reagent, and 0.1 mL of this reagent was added into 5 mL of the diluted electrolyte collected after the electrolysis. The mixture was placed at room temperature for 20 min, and the UV-visible spectrum of the solution was immediately collected; the intensity of the absorption peak located at 537 nm was recorded. The calibration curve for quantifying nitrite ions was obtained by using 5 mL of $NaNO_2$ aqueous solution with a known concentration to replace the diluted electrolyte.

S2. SEM images and pore size distributions

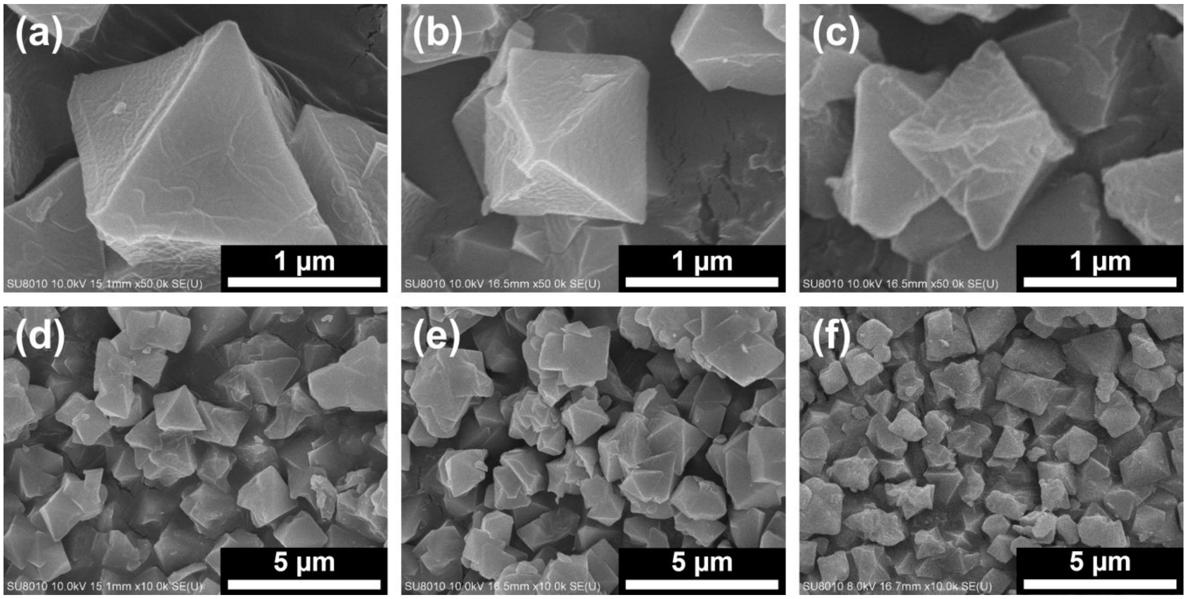

Figure S1. High-magnification SEM images of (a) MOF-808, (b) SO_3-MOF-808, and (c) TMA-MOF-808. Low-magnification SEM images of (d) MOF-808, (e) SO_3-MOF-808, and (f) TMA-MOF-808.

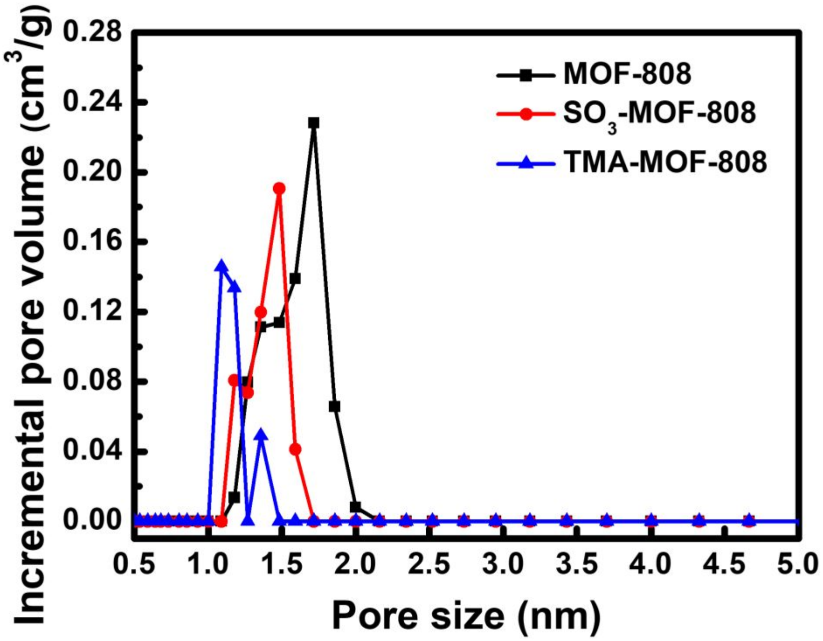

Figure S2. DFT pore size distributions of MOF-808, SO_3-MOF-808, and TMA-MOF-808, calculated from their isotherms with the use of the ASAP 2020 system (Micromeritics) and the carbon slit-pore DFT model.

S3. CV analysis for gauging ECSA

To gauge the non-Faradaic current of all modified electrodes with copper, CV experiments at slow scan rates were conducted between +0.24 V and +0.29 V *vs*. SHE. All CV curves displayed in Figure S3 were scanned from +0.29 V to +0.24 V first and thereafter scanned back. It should be noticed that at an applied potential more negative than +0.24 V, an obvious cathodic Faradaic current would be generated. In addition, the oxidation of copper starts at around +0.27 V, as revealed from the shape of CV curves in Figure S3. A roughly rectangular region, which represents the non-Faradaic process occurring on the electrode, can be observed in all CV curves between +0.24 V and +0.26 V. Therefore, values of Δi/2 plotted in Figure 3(c) of the main text were extracted from values of current density recorded at +0.25 V in CV curves shown in Figure S3.

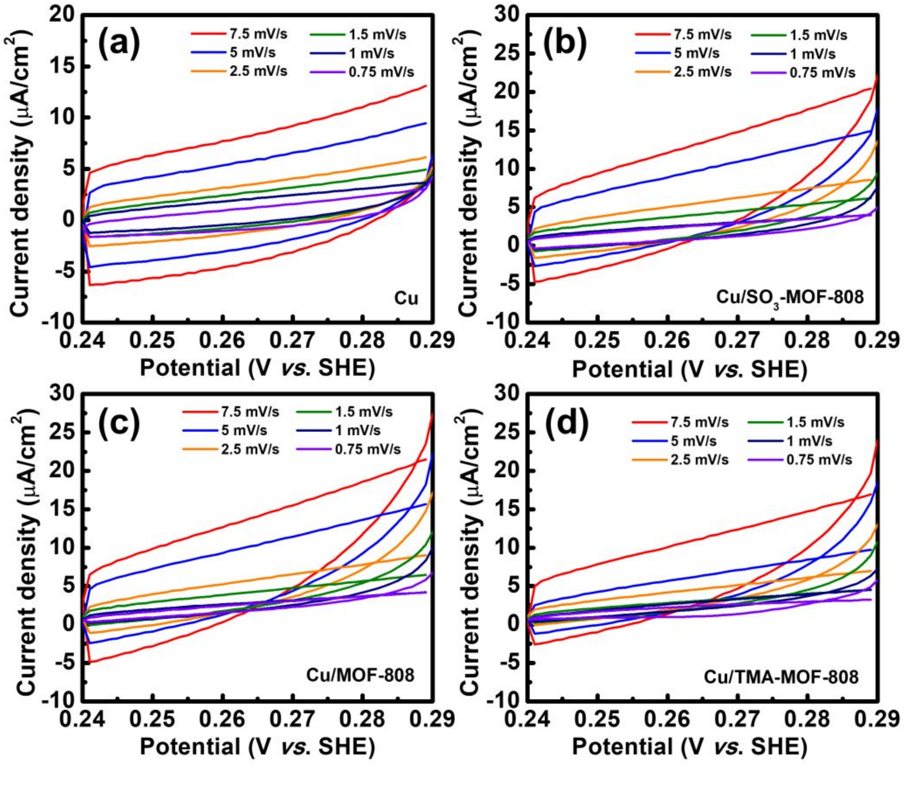

Figure S3. CV curves of modified electrodes of (a) Cu, (b) Cu/SO_3-MOF-808, (c) Cu/MOF-808, and (d) Cu/TMA-MOF-808, measured in aqueous electrolytes containing 0.5 M of Na_2SO_4 and 0.5 M of $NaNO_3$ at various scan rates.

S4. Chemical stability of MOF thin films after electrolysis

Cu/SO_3-MOF-808 modified electrodes were subjected to electrolytic experiments in aqueous electrolytes containing 0.5 M of Na_2SO_4 and 0.5 M of $NaNO_3$ at various applied potentials for 1 h. After each electrolytic experiment, the modified electrode was removed from the electrolyte and rinsed three times by soaking in 10 mL of water, with the immersing period of 10 min for each time to fully remove Na_2SO_4 and $NaNO_3$ from the electrode. The electrode was then transferred into 10 mL of acetone for solvent exchange. The solvent-exchange process was performed three times with the fresh acetone with immersing periods of 2 h, overnight, and 2 h in order. The electrode was then dried at 60 °C. The insulating tape was thereafter removed from the modified electrode, and the electrode was subjected to GIXRD measurements. GIXRD pattern of the fresh Cu/SO_3-MOF-808 modified electrode was also measured for comparison; see Figure S4(a). Diffraction peaks of MOF-808 are clearly present in patterns of all electrodes except for the electrode after the electrolysis at -1.29 V. Findings here indicate that the electrolysis at -1.29 V causes a strong change in pH, resulting in the structural degradation of the MOF coating.

In addition, the Cu/SO_3-MOF-808 modified electrode after the electrolysis at -1.19 V and activation processes mentioned above was immersed in a few drops of D_2SO_4 in a glass vial to digest the MOF, and after the sonication for 10 min, 1 mL of DMSO-d_6 was added to prepare the solution for NMR experiments. As revealed in Figure S4(b), both signals from the trimistic acid linker (around 8.2-8.3 ppm) and sulfoacetic acid (3.5 ppm) can be observed, and a loading of 1.1 sulfoacetic acid per node was obtained. This loading is almost the same as that of the fresh SO_3-MOF-808 powder (1.2 ligand per node), indicating the chemical stability of the MOF coating after the electrolysis in the electrolyte containing 0.5 M of Na_2SO_4 and 0.5 M of $NaNO_3$ at -1.19 V *vs*. SHE. Furthermore, the morphology of SO_3-MOF-808 composed of octahedral crystals is unchanged after the electrolysis at -1.19 V, as revealed in SEM images shown in Figure S4(c-d).

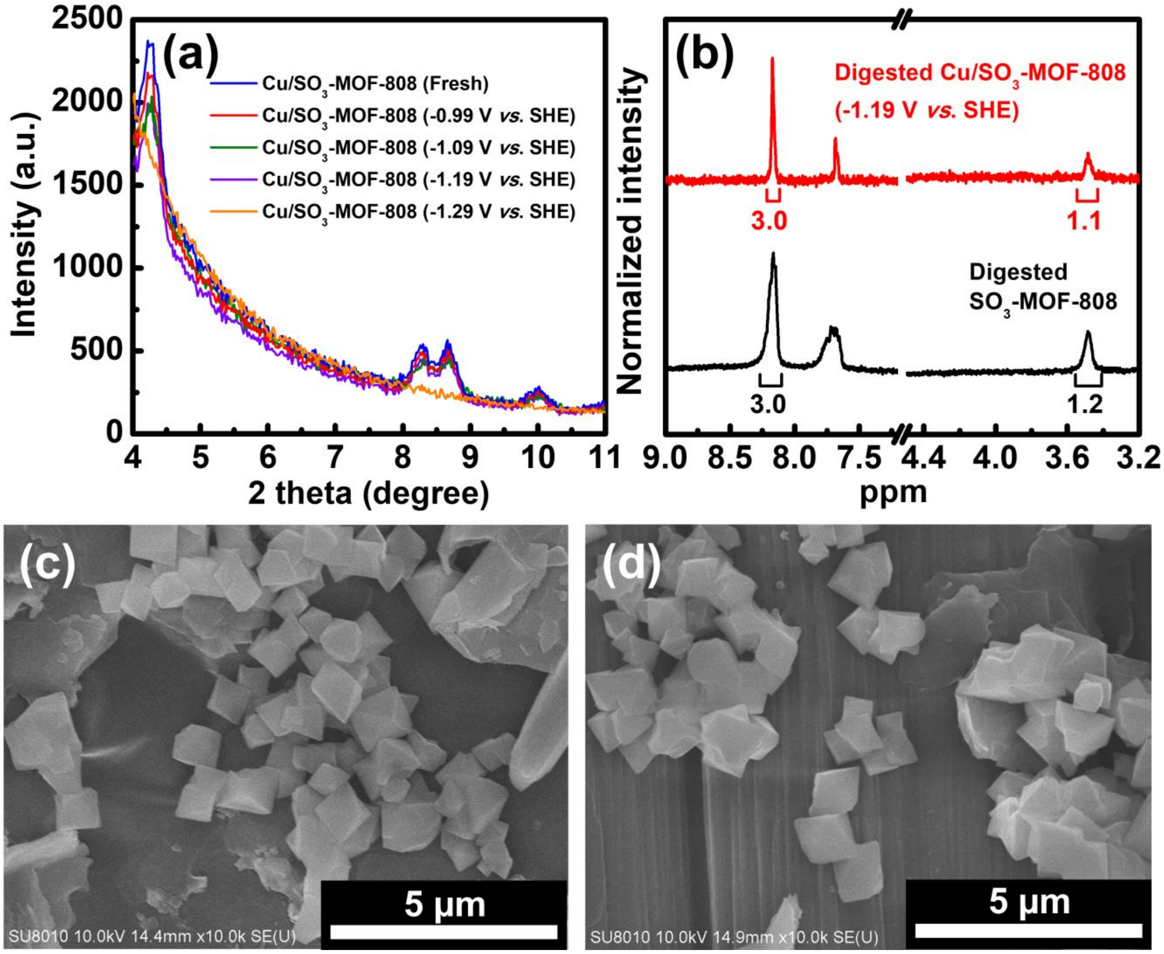

Figure S4. (a) GIXRD patterns of Cu/SO_3-MOF-808 electrodes before and after electrolytic experiments at various applied potentials in electrolytes containing 0.5 M of Na_2SO_4 and 0.5 M of $NaNO_3$ for 1 h. (b) NMR spectra of the digested SO_3-MOF-808 and the digested thin film from the Cu/SO_3-MOF-808 electrode after the electrolysis at -1.19 V *vs*. SHE in the electrolyte containing 0.5 M of Na_2SO_4 and 0.5 M of $NaNO_3$ for 1 h. SEM images of Cu/SO_3-MOF-808 electrodes collected in regions with MOF crystals (c) before and (d) after the electrolysis at -1.19 V *vs*. SHE in the electrolyte containing 0.5 M of Na_2SO_4 and 0.5 M of $NaNO_3$ for 1 h.

S5. Chronoamperometric and UV-visible data (0.5 M of nitrate)

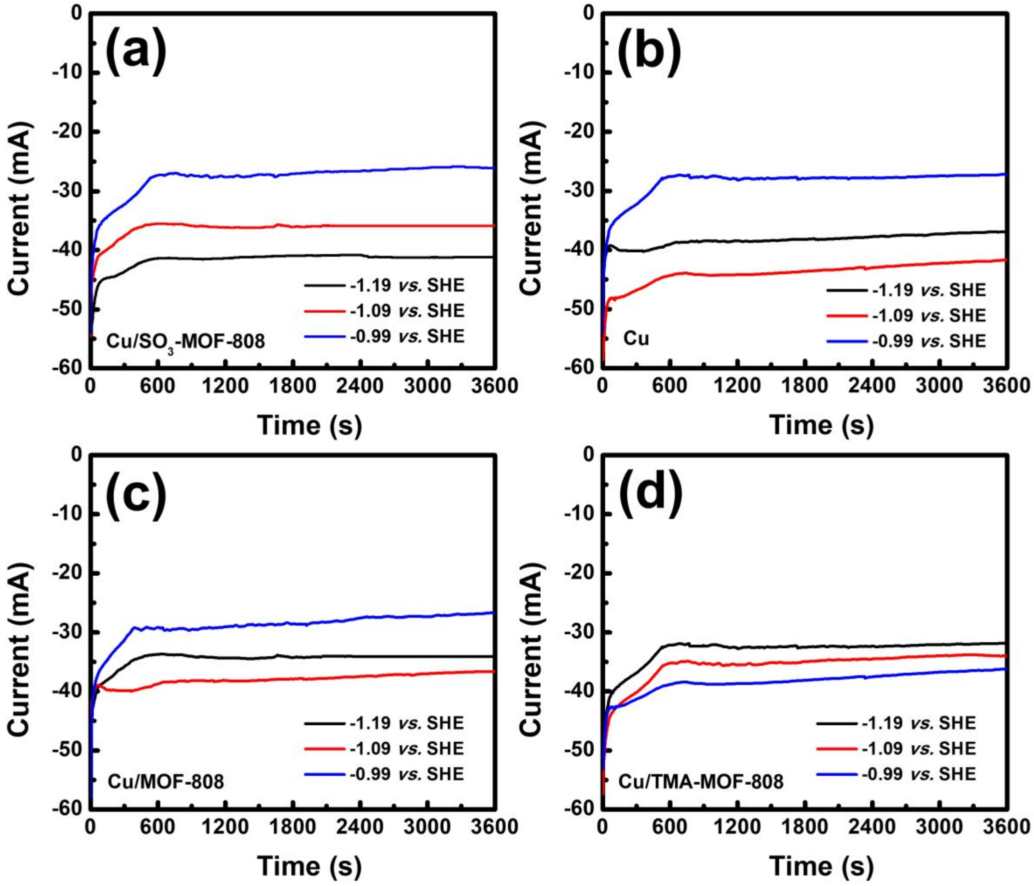

Figure S5. Representative chronoamperometric data of (a) Cu/SO_3-MOF-808, (b) Cu, (c) Cu/MOF-808, and (d) Cu/TMA-MOF-808, recorded during the electrolytic experiments at various applied potentials in electrolytes containing 0.5 M of Na_2SO_4 and 0.5 M of $NaNO_3$.

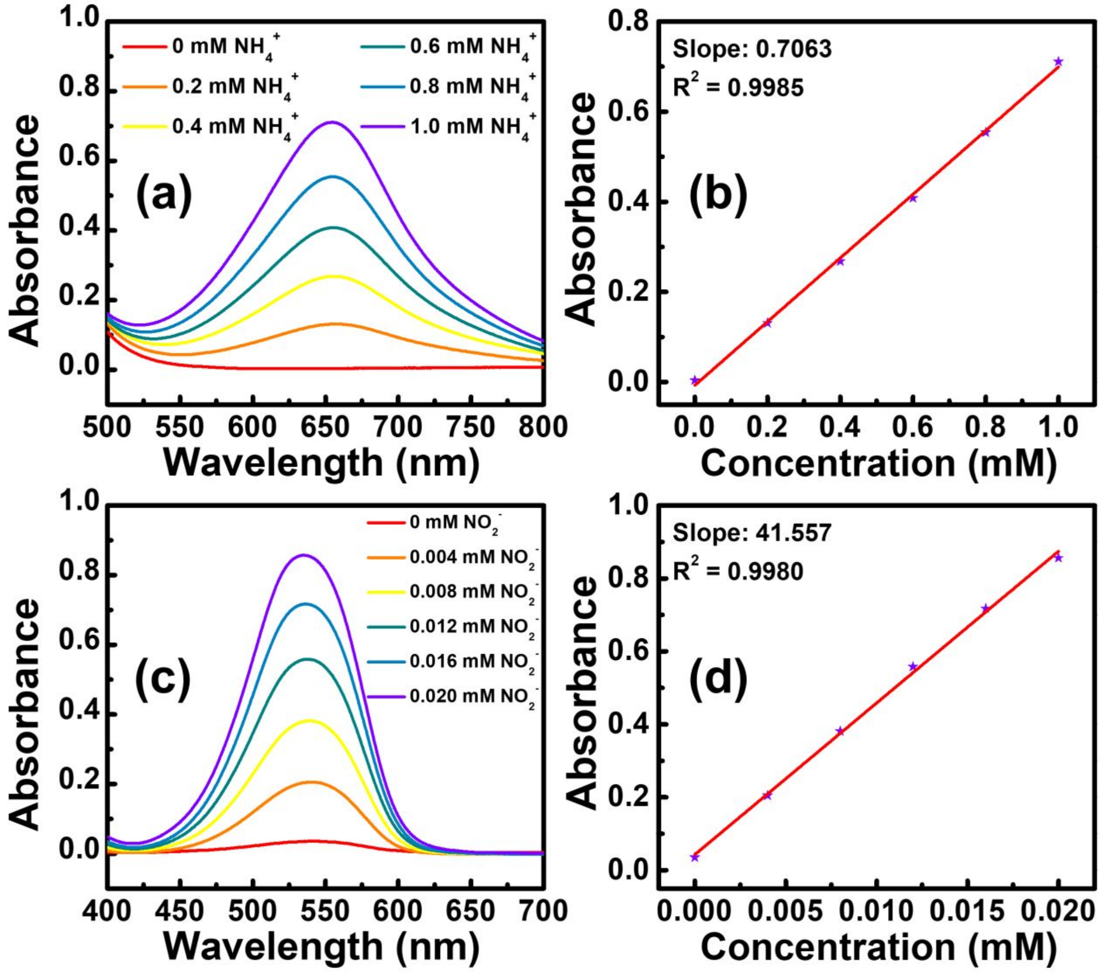

Figure S6. UV-visible data of standard solutions containing (a) ammonium ions and (c) nitrite ions. See experimental details in section S1. Calibration curves for product analysis are shown in (b) and (d).

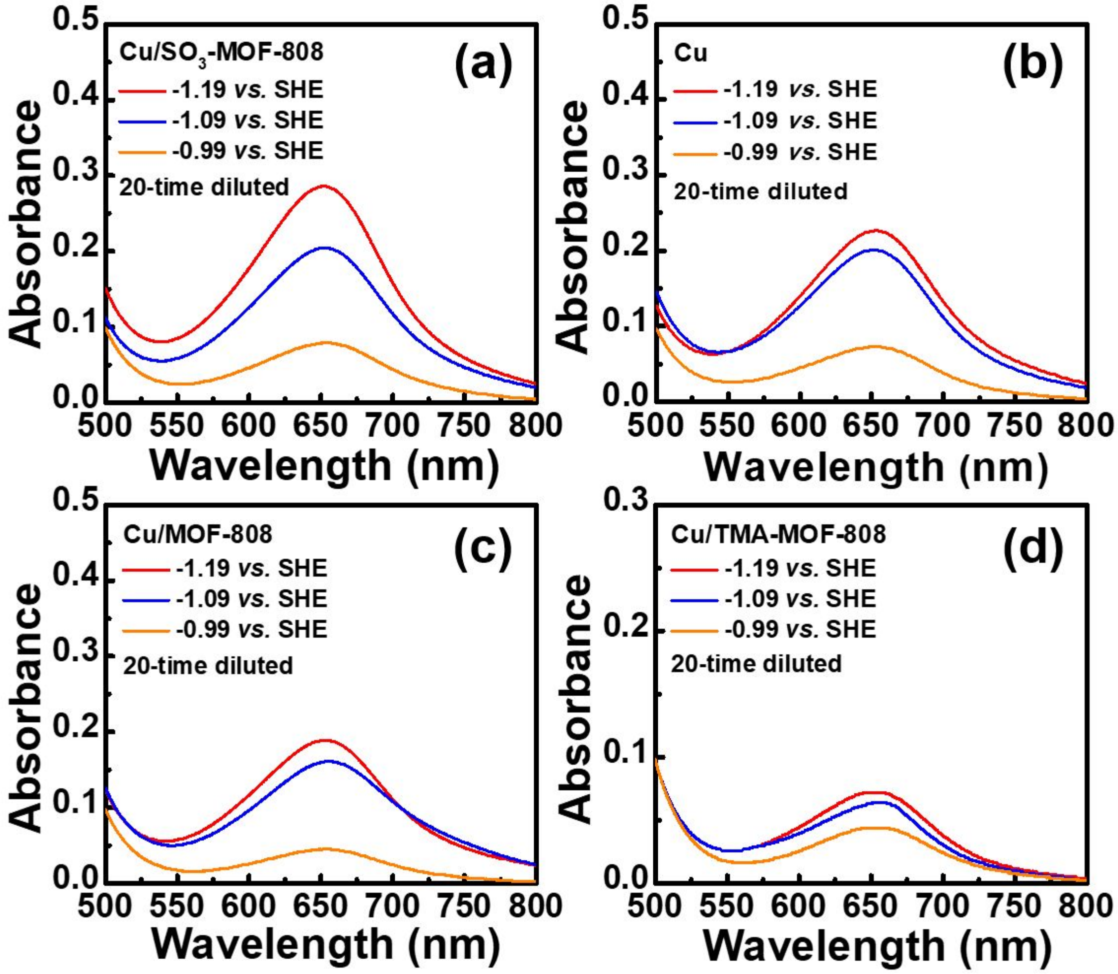

Figure S7. Representative UV-visible data of electrolytes collected after various electrolytic experiments in electrolytes containing 0.5 M of Na_2SO_4 and 0.5 M of $NaNO_3$, aiming for quantifying ammonium. See experimental details in section S1. (a) Cu/SO_3-MOF-808, (b) Cu, (c) Cu/MOF-808, and (d) Cu/TMA-MOF-808 were used for electrolysis. Each electrolyte was twentyfold diluted before the measurement.

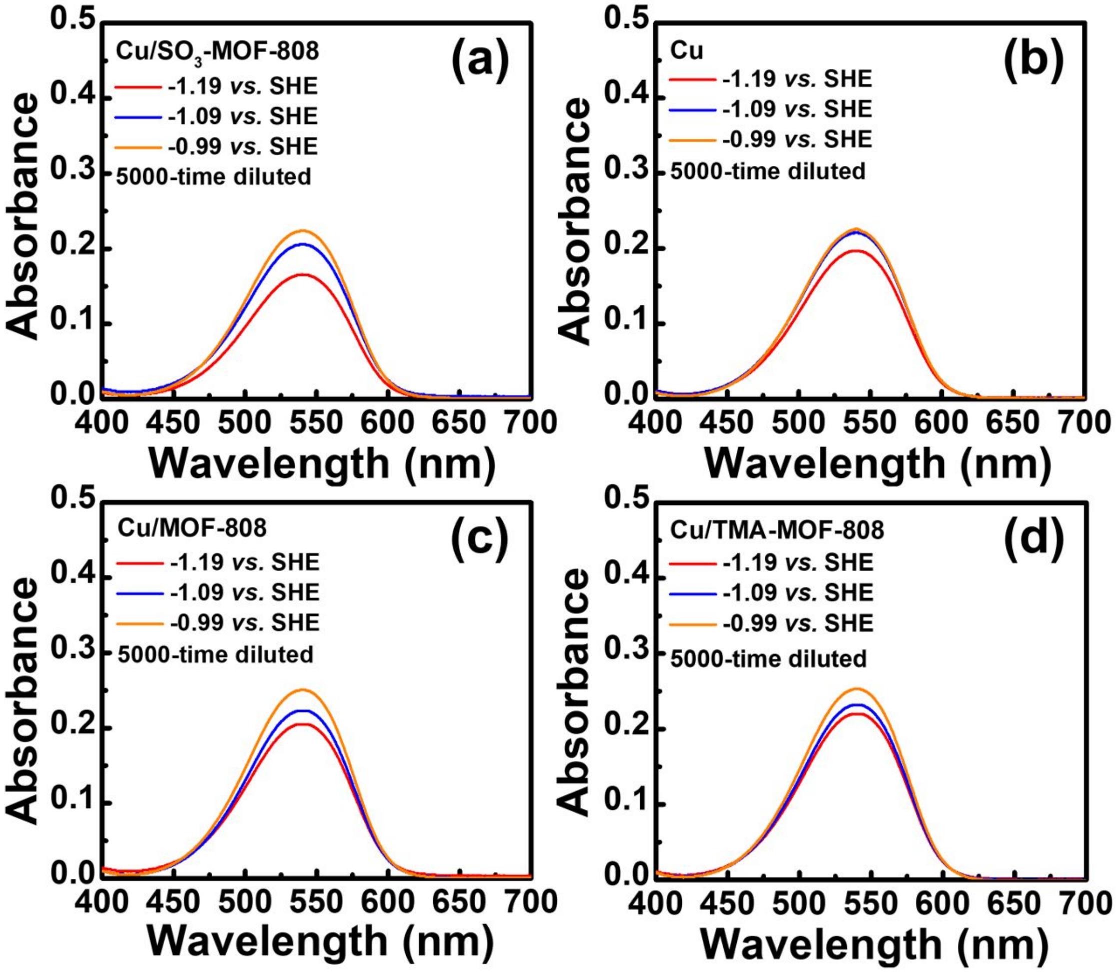

Figure S8. Representative UV-visible data of electrolytes collected after various electrolytic experiments in electrolytes containing 0.5 M of Na_2SO_4 and 0.5 M of $NaNO_3$, aiming for quantifying nitrite. See experimental details in section S1. (a) Cu/SO_3-MOF-808, (b) Cu, (c) Cu/MOF-808, and (d) Cu/TMA-MOF-808 were used for electrolysis. Each electrolyte was 5000-fold diluted before the measurement.

S6. Calculation of Faradaic efficiencies (FE) and selectivity

From the calibration curves shown in Figure S6 and UV-visible spectra of diluted electrolytes after electrolytic experiments, concentrations of ammonium and nitrite in each electrolyte in the compartment of working electrode (15 mL) were obtained. By assuming that all produced ammonia was dissolved as ammonium ions, the production rate of ammonia (in mmol/cm^2-h) can be calculated from the concentration of ammonia (C_{NH3}, in mM) by the following equation,

$$\text{Production rate of ammonia} = \frac{C_{NH3} \times V}{t \times A} \quad \text{(S1)}$$

where V is the volume of the electrolyte (0.015 L), t is the time for electrolysis (1 h), and A is the area of the electrode (1 cm^2). FE for the production of ammonia and FE for the production of nitrite were then calculated by using the following two equations with the use of C_{NH3} and the concentration of nitrite (C_{NO2}, in mM) in the electrolyte,

$$\text{FE for ammonia production} = \frac{8 \times F \times C_{NH3} \times V}{Q \times 1000} \times 100\% \quad \text{(S2)}$$

$$\text{FE for nitrite production} = \frac{2 \times F \times C_{NO2} \times V}{Q \times 1000} \times 100\% \quad \text{(S3)}$$

where F is the Faraday constant (96485 C/mol) and Q is the integrated charge from the chronoamperometric data (in C).

It has been widely reported that for copper-based electrocatalysts, the formation of other products in addition to ammonia and nitrite from NO_3RR is negligible,[1-3] and HER is the major side reaction for the rest of FE. Thus, the selectivity of NO_3RR toward ammonia was calculated by the following equation.

$$\text{Selectivity of } NO_3\text{RR toward ammonia} = \frac{C_{NH3}}{C_{NH3} + C_{NO2}} \times 100\% \quad \text{(S4)}$$

S7. Additional electrolytic data (0.5 M of nitrate)

Table S1. FE and selectivity of each copper-based modified electrode from electrolytic experiments at various applied potentials, performed with 0.5 M of nitrate ions in electrolytes.

Electrode	Electrolyte	Potential (*vs*. SHE)	NH_3 FE (%)	NO_2^- FE (%)	NH_3 Selectivity (%)	NO_2^- Selectivity (%)
Cu/SO_3-MOF-808	0.5 M Na_2SO_4/ 0.5 M $NaNO_3$	-1.19	64.2	28.3	36.1	63.9
		-1.09	53.3	43.4	23.5	76.5
		-0.99	28.3	63.2	10.0	89.9
Cu/MOF-808		-1.19	51.8	45.4	22.2	77.8
		-1.09	40.3	45.8	18.0	82.0
		-0.99	16.4	69.6	5.6	94.4
Cu		-1.19	55.5	38.9	26.3	73.7
		-1.09	43.2	39.3	21.6	78.4
		-0.99	25.8	62.5	9.3	90.7
Cu/TMA-MOF-808		-1.19	21.8	51.7	9.5	90.5
		-1.09	18.1	51.1	8.1	91.9
		-0.99	12.2	53.0	5.4	94.6

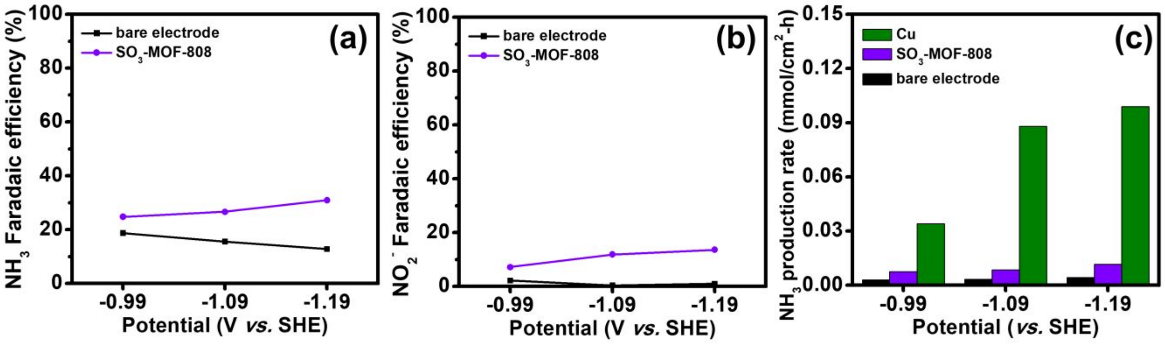

Figure S9. (a) FE for ammonia production, (b) FE for nitrite production, and (c) ammonia production rates of bare carbon papers and SO_3-MOF-808-modified carbon papers, obtained from one-hour electrolytic experiments with electrolytes containing 0.5 M of Na_2SO_4 and 0.5 M of $NaNO_3$. Data of Cu-modified carbon papers are also plotted in (c).

S8. Effect of the MOF loading on top of the Cu electrode

Various amounts of SO_3-MOF-808 were then deposited on top of Cu electrodes in order to optimize the loading of the MOF coating. It should be noticed that since the porous carbon paper was used as the underlying current collector and the electrocatalytic Cu was electrodeposited on the carbon paper, the surface of the Cu-modified electrode possesses needle-like crystals of electrodeposited copper less uniformly covering on the fibrous carbon paper (as shown in all subfigures in Figure S10). Thus, by simply performing the drop-casting method to deposit the MOF coating, SO_3-MOF-808 crystals can only be randomly deposited on top of the highly rough surface of Cu. As revealed in SEM images shown in Figure S10, by adjusting the MOF loading from 0.144 to 0.864 mg/cm^2, the coverage of MOF crystals on the rough surface increases, but more aggregates of MOF crystals can also be observed; the thickness of each MOF coating is thus ill-defined. These Cu-modified electrodes with various loadings of SO_3-MOF-808 were then subjected to electrolytic experiments at -1.19 V *vs*. SHE in the presence of 0.5 M of nitrate ions, and as revealed in Figure S11, the electrode with 0.288 mg/cm^2 of MOF exhibits the optimal performance. With a small MOF loading, the low coverage of MOF crystals on the Cu surface reduces the proton-enriching effect, while the high MOF loading may retard the overall mass transfer of all species from and to the electrode, especially at such a large operating current density. To achieve the proton-enriching effect with a high mass loading of MOF and a fully covered coating, frameworks with larger pores may be required. Herein, the optimal MOF loading, *i.e.*, 0.288 mg/cm^2, was selected throughout the rest of this study.

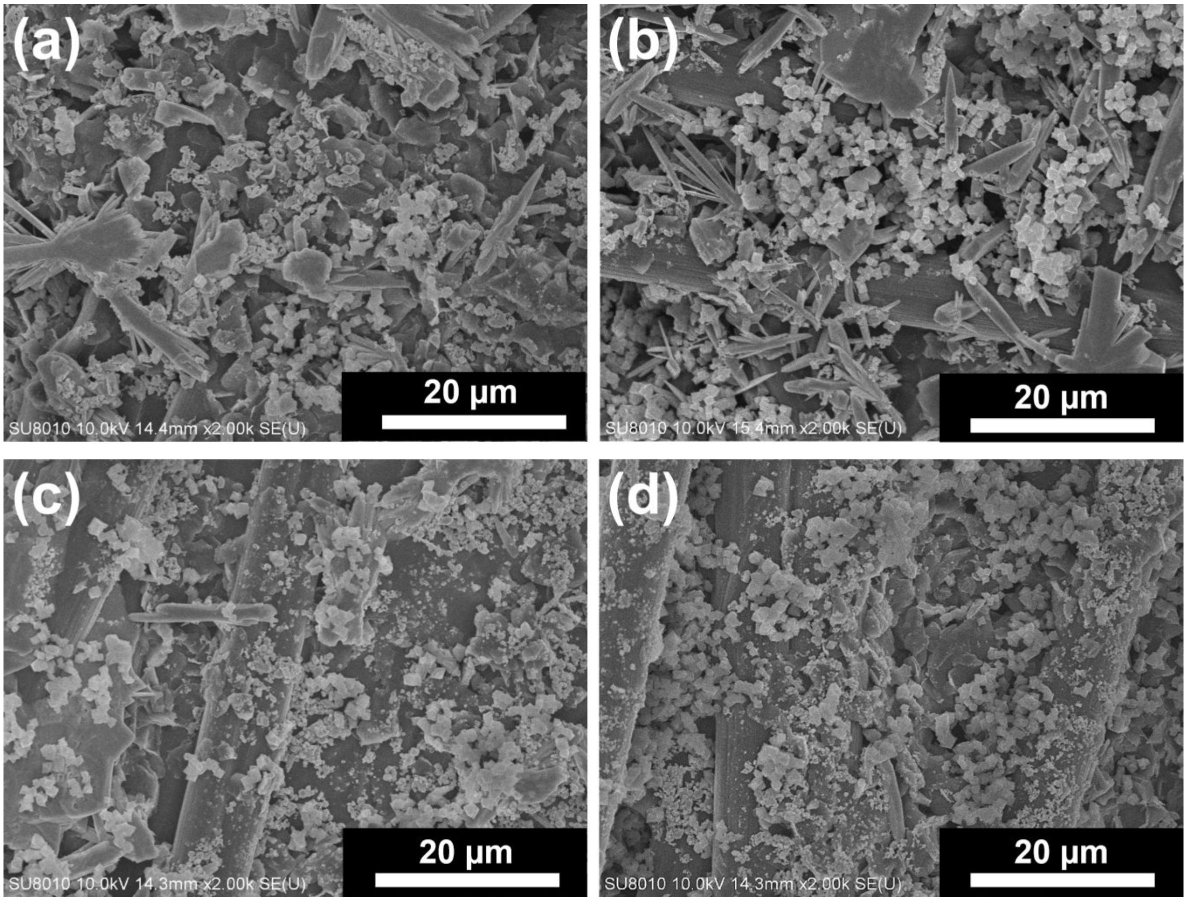

Figure S10. Representative SEM images of Cu/SO_3-MOF-808 electrodes with various loadings of SO_3-MOF-808: (a) 0.144, (b) 0.288, (c) 0.576, and (d) 0.864 mg/cm^2.

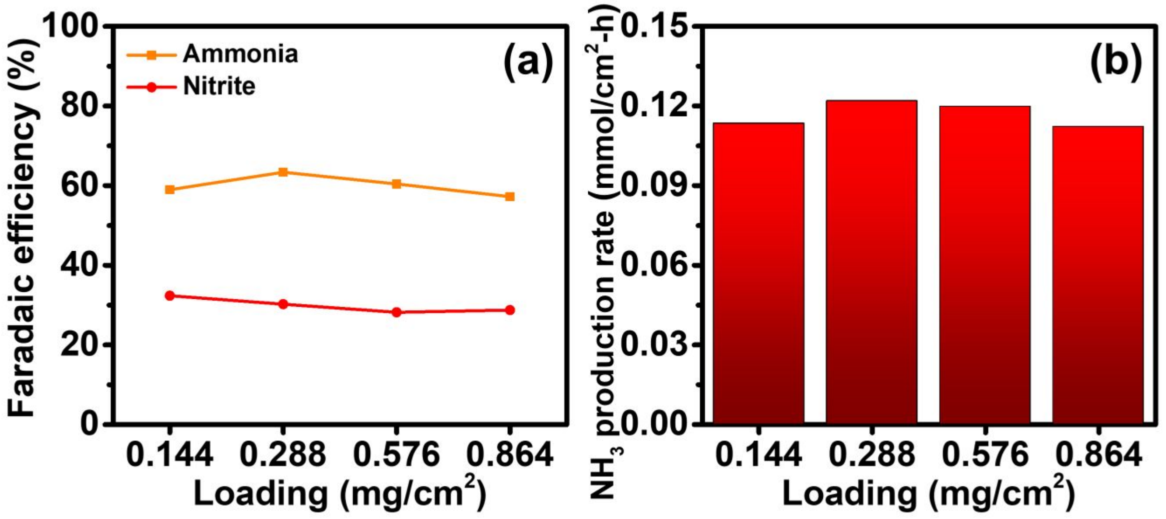

Figure S11. (a) FE of NO_3RR and (b) ammonia production rates of Cu/SO_3-MOF-808 electrodes with various mass loadings of SO_3-MOF-808 coatings. All data were obtained from one-hour electrolytic experiments at -1.19 V *vs*. SHE with electrolytes containing 0.5 M of Na_2SO_4 and 0.5 M of $NaNO_3$.

S9. Chronoamperometric and UV-visible data (various nitrate conc.)

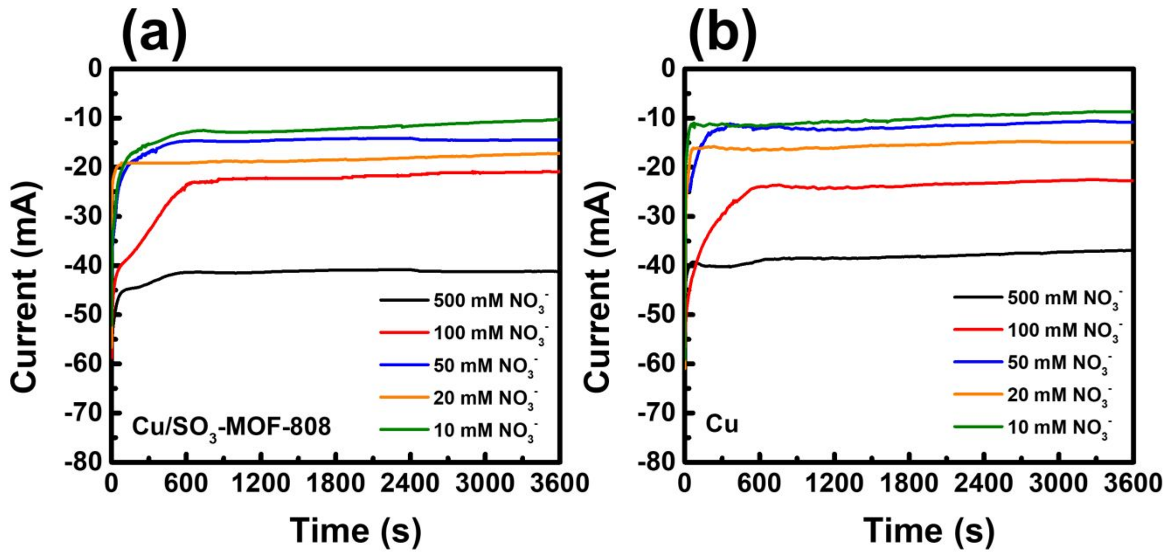

Figure S12. Representative chronoamperometric data of (a) Cu/SO_3-MOF-808 and (b) Cu, recorded during the electrolytic experiments at -1.19 V *vs*. SHE in electrolytes containing 0.5 M of Na_2SO_4 and various concentrations of $NaNO_3$.

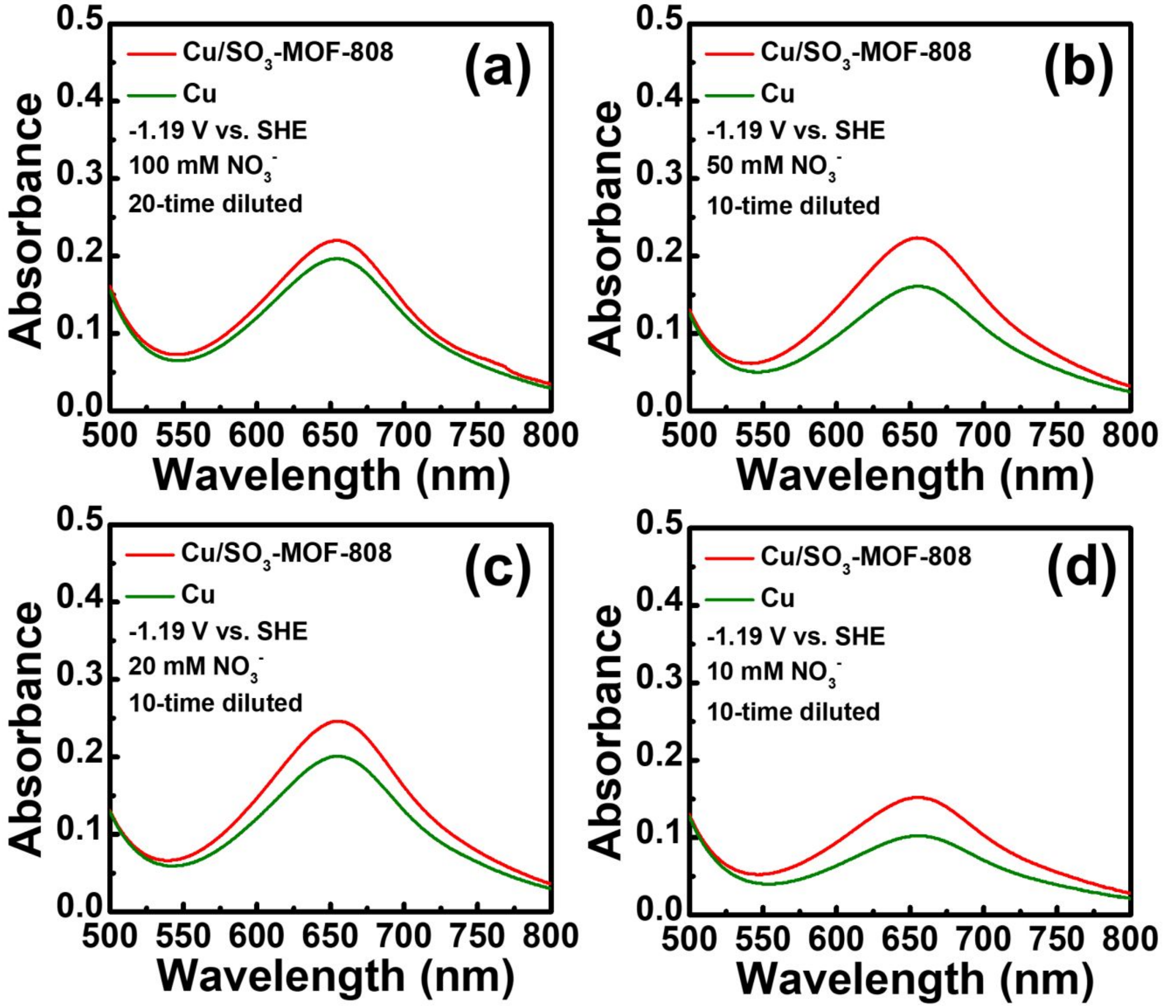

Figure S13. Representative UV-visible data of electrolytes collected after various electrolytic experiments in electrolytes containing 0.5 M of Na_2SO_4 and (a) 100 mM of $NaNO_3$, (b) 50 mM of $NaNO_3$, (c) 20 mM of $NaNO_3$, and (d) 10 mM of $NaNO_3$, aiming for quantifying ammonium. See experimental details in section S1. Different degrees of dilution were performed to the original electrolytes to reach the detecting range before measurements, as indicated in each subfigure.

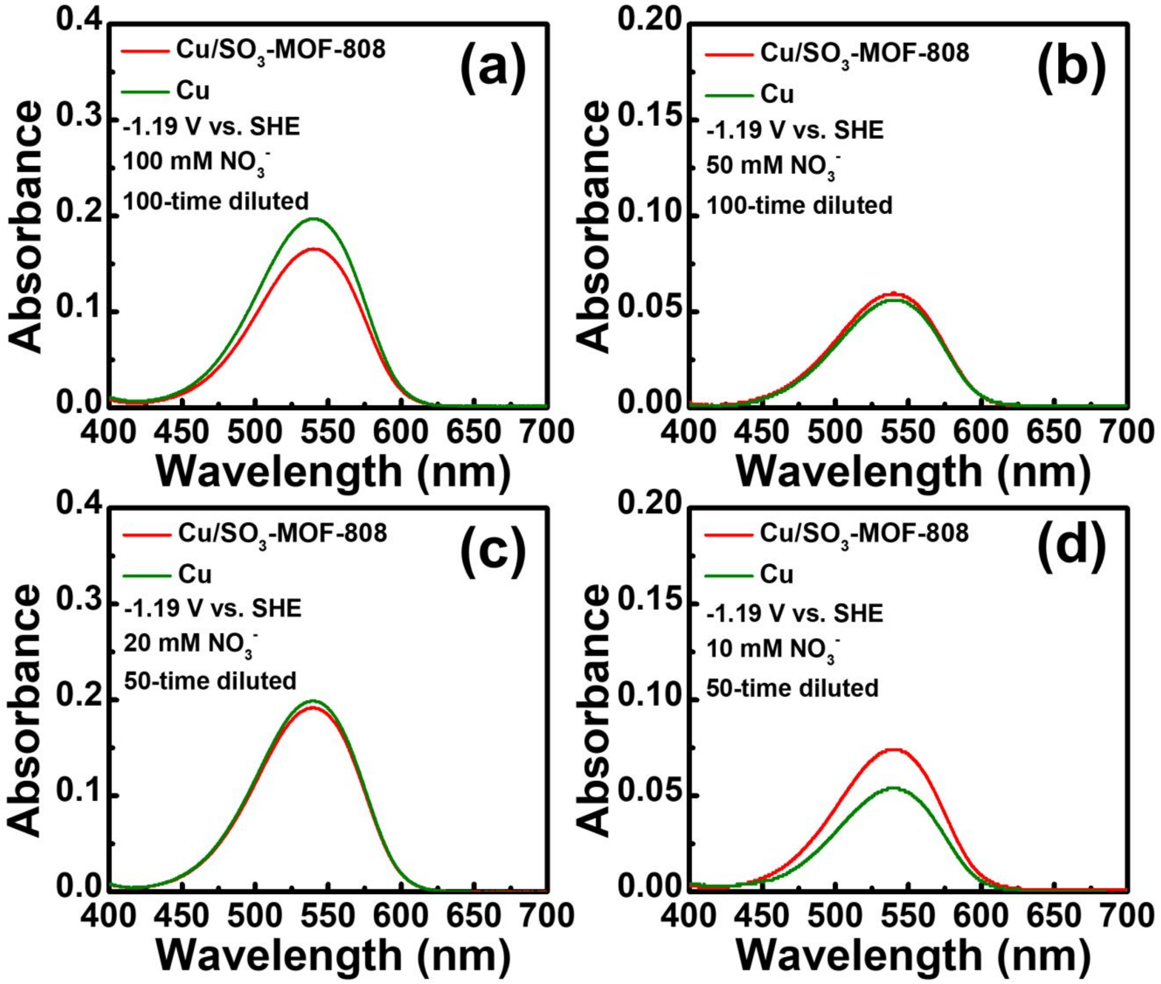

Figure S14. Representative UV-visible data of electrolytes collected after various electrolytic experiments in electrolytes containing 0.5 M of Na_2SO_4 and (a) 100 mM of $NaNO_3$, (b) 50 mM of $NaNO_3$, (c) 20 mM of $NaNO_3$, and (d) 10 mM of $NaNO_3$, aiming for quantifying nitrite. See experimental details in section S1. Different degrees of dilution were performed to the original electrolytes to reach the detecting range before measurements, as indicated in each subfigure.

Table S2. FE and selectivity of Cu/SO_3-MOF-808 and pristine Cu electrodes obtained from electrolytic experiments at -1.19 V *vs*. SHE, performed with various concentrations of nitrate ions in electrolytes.

Electrode	Concentration of nitrate (mM)	Potential (*vs*. SHE)	FE for NH_3 (%)	FE for NO_2^- (%)	NH_3 Selectivity (%)
Cu/SO_3-MOF-808	500	-1.19	64.2	28.3	36.1
	100		87.5	1.0	95.6
	50		69.5	0.2	98.8
	20		62.2	0.8	95.2
	10		57.2	0.2	98.4
Cu	500		55.5	38.9	26.3
	100		75.0	1.2	94.0
	50		63.8	0.2	98.7
	20		60.6	1.0	94.0
	10		48.7	0.1	99.2

S10. Chronoamperometric and UV-visible data of Nafion-cast electrodes

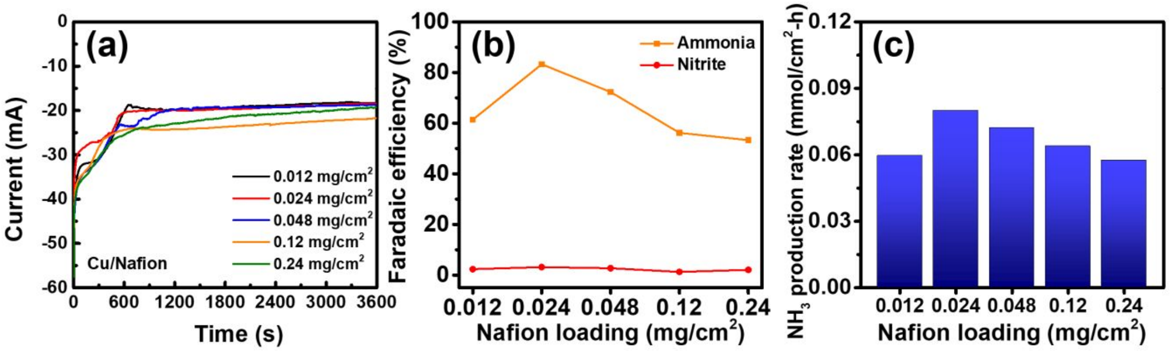

Figure S15. (a) Representative chronoamperometric data of Cu electrodes with various loadings of Nafion coatings, recorded during the electrolytic experiments at -1.19 V *vs*. SHE in electrolytes containing 0.5 M of Na_2SO_4 and 100 mM of $NaNO_3$. (b) FE for ammonia and nitrite and (c) production rates of ammonia, obtained from the electrolytic measurements shown in (a).

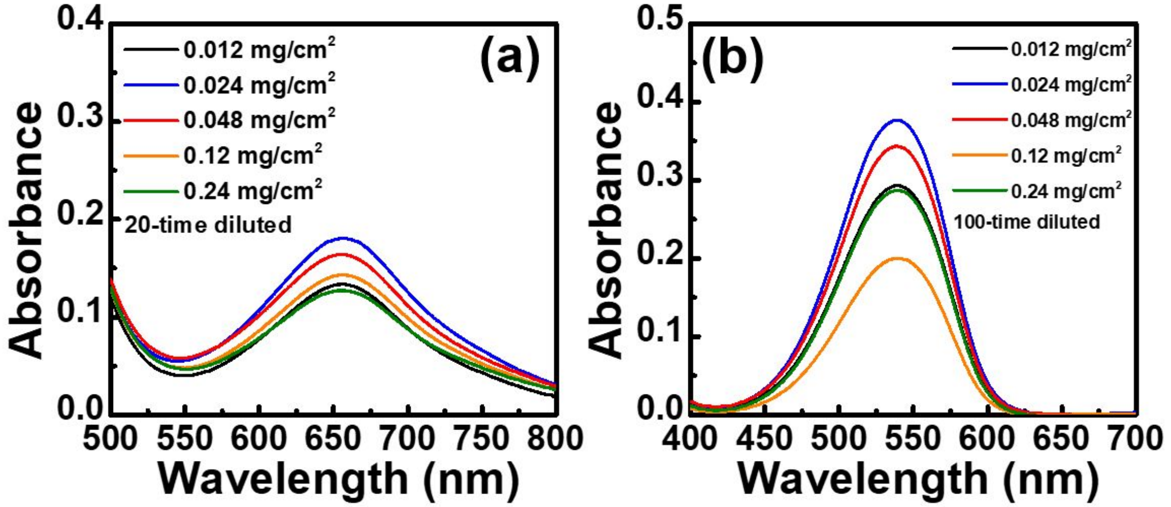

Figure S16. Representative UV-visible data of electrolytes collected after various electrolytic experiments with Cu electrodes with various loadings of Nafion coatings in electrolytes containing 0.5 M of Na_2SO_4 and 100 mM of $NaNO_3$. Data for quantifying ammonia and nitrite are shown in (a) and (b), respectively. Different degrees of dilution were performed to the original electrolytes to reach the detecting range before measurements, as indicated in each subfigure.

Table S3. FE and selectivity of Cu/SO_3-MOF-808, Cu/Nafion, and pristine Cu electrodes obtained from electrolytic experiments at -1.19 V *vs*. SHE, performed with 100 mM of nitrate ions in electrolytes.

Electrode	Electrolyte	Potential (*vs*. SHE)	FE for NH_3 (%)	FE for NO_2^- (%)	NH_3 Selectivity (%)
Cu/SO_3-MOF-808	0.5 M Na_2SO_4 / 100 mM $NaNO_3$	-1.19	87.5	1.0	95.6
Cu			75.0	1.2	94.0
Cu/Nafion			83.3	3.1	86.9

S11. Comparison to performances in the literature

Table S4. Comparison to a partial list of recent studies in the field of NO_3RR.

Material	Electrolyte	NH_3 yield rate (mmol/h-mg_{cat})	FE for NH_3 (%)	NH_3 Selectivity (%)	Reference
Cu/SO_3-MOF-808	100 mM $NaNO_3$ + 0.5 M Na_2SO_4	0.383	87.5	95.6	
Cu	100 mM $NaNO_3$ + 0.5 M Na_2SO_4	0.345	75.0	93.9	This work
Cu/Nafion	100 mM $NaNO_3$ + 0.5 M Na_2SO_4	0.318	83.3	86.9	
Cu/Ce_2O_3	100 mM NO_3^- + 0.5 M Na_2SO_4	0.386	73	-	[4]
Fe SAC [a]	100 mM KNO_3 + 1 M KOH	0.309	75	69	[2]
$Cu_{30\%}$@NHC [b]	100 mM KNO_3 + 0.1 M Na_2SO_4	0.213	77.05	-	[3]
Cu-N-C SAC [c]	100 mM KNO_3 + 1 M KOH	0.199	84.7	-	[5]
Cu SAGs [d]	20 mM $NaNO_3$ + 0.1 M PBS [j]	0.065	78	-	[6]
Cu@Cu_2O MSs [e]	500 ppm NO_3^- ($NaNO_3$) + 0.1 M Na_2SO_4	0.328	80.57	84.63	[7]

Cu@CuHHTP	500 ppm NO_3^- ($NaNO_3$) + 0.5 M Na_2SO_4	0.216	67.55	96.84	[8]
Cu/Cu_2O NWAs [f]	200 ppm NO_3^- ($NaNO_3$) + 0.5 M Na_2SO_4	-	95.8	81.2	[1]
UiO-CuZn	200 ppm NO_3^- ($NaNO_3$) + 0.5 M Na_2SO_4	0.00023	91.4	95.2	[9]
CuCl_BEF [g]	100 ppm NO_3^- (KNO_3) + 0.5 M Na_2SO_4	0.097	44.7	98.6	[10]
Cu@C	1 mM KNO_3 + 1 M KOH	0.028	72	-	[11]
Plasma-treated Cu_2O	50 ppm NO_3^- ($NaNO_3$) + 0.5 M Na_2SO_4	0.083	89.5	85.7	[12]
Cu nanotubes	50 ppm NO_3^- (KNO_3) + 0.5 M K_2SO_4	0.046	85.7	86.2	[13]
Cu nanoplates [h]	50 ppm NO_3^- (KNO_3) + 0.5 M K_2SO_4	0.046	85.47	81.99	[14]
$Cu@Cu_{2+1}O$ NWs [i]	50 ppm NO_3^- (KNO_3) + 0.5 M K_2SO_4	0.034	87.07	76	[15]

[a] Fe SAC: Fe single-atom catalyst
[b] $Cu_{30\%}$@NHC: Cu nanoparticles on porous nitrogen-doped hexagonal carbon nanorods
[c] Cu-N-C SAC: Cu ions doped into porous nitrogen-doped carbon single-atom catalysts
[d] Cu SAGs: Cu single-atom gels
[e] $Cu@Cu_2O$ MSs: $Cu@Cu_2O$ microspheres
[f] Cu/Cu_2O NWAs: Cu/Cu_2O nanowire arrays
[g] CuCl_BEF: CuCl-based catalyst with a built-in electric field
[h] Cu nanoplates: Defect-rich metallic Cu nanoplates
[i] $Cu@Cu_{2+1}O$ NWs: $Cu@Cu_{2+1}O$ core-sheath nanowires
[j] PBS: Phosphate buffer solution

S12. References

1. Wang, Y.; Zhou, W.; Jia, R.; Yu, Y.; Zhang, B., Unveiling the Activity Origin of a Copper-Based Electrocatalyst for Selective Nitrate Reduction to Ammonia. *Angew. Chem. Int. Ed.* **2020,** *59*, 5350-5354.

2. Wu, Z.-Y.; Karamad, M.; Yong, X.; Huang, Q.; Cullen, D. A.; Zhu, P.; Xia, C.; Xiao, Q.; Shakouri, M.; Chen, F.-Y.; Kim, J. Y.; Xia, Y.; Heck, K.; Hu, Y.; Wong, M. S.; Li, Q.; Gates, I.; Siahrostami, S.; Wang, H., Electrochemical Ammonia Synthesis *via* Nitrate Reduction on Fe Single Atom Catalyst. *Nat. Commun.* **2021,** *12*, 2870.

3. Zhang, J.; Chen, C.; Zhang, R.; Wang, X.; Wei, Y.; Sun, M.; Liu, Z.; Ge, R.; Ma, M.; Tian, J., Size-Induced D Band Center Upshift of Copper for Efficient Nitrate Reduction to Ammonia. *J. Colloid Interface Sci.* **2024,** *658*, 934-942.

4. Li, D.; Wang, F.; Mao, J., Surface-Reconstructed Copper Foil Free-Standing Electrode with Nanoflower Cu/Ce_2O_3 by in Situ Electrodeposition Reduction for Electrocatalytic Nitrate Reduction to Ammonia. *Inorg. Chem.* **2023,** *62*, 16283-16287.

5. Yang, J.; Qi, H.; Li, A.; Liu, X.; Yang, X.; Zhang, S.; Zhao, Q.; Jiang, Q.; Su, Y.; Zhang, L.; Li, J.-F.; Tian, Z.-Q.; Liu, W.; Wang, A.; Zhang, T., Potential-Driven Restructuring of Cu Single Atoms to Nanoparticles for Boosting the Electrochemical Reduction of Nitrate to Ammonia. *J. Am. Chem. Soc.* **2022,** *144*, 12062-12071.

6. Li, P.; Li, R.; Liu, Y.; Xie, M.; Jin, Z.; Yu, G., Pulsed Nitrate-to-Ammonia Electroreduction Facilitated by Tandem Catalysis of Nitrite Intermediates. *J. Am. Chem. Soc.* **2023,** *145*, 6471-6479.

7. Jiang, M.; Zhu, Q.; Song, X.; Gu, Y.; Zhang, P.; Li, C.; Cui, J.; Ma, J.; Tie, Z.; Jin, Z., Batch-Scale Synthesis of Nanoparticle-Agminated Three-Dimensional Porous Cu@Cu_2O Microspheres for Highly Selective Electrocatalysis of Nitrate to Ammonia. *Environ. Sci. Technol.* **2022,** *56*, 10299-10307.

8. Zhu, X.; Huang, H.; Zhang, H.; Zhang, Y.; Shi, P.; Qu, K.; Cheng, S.-B.; Wang, A.-L.; Lu, Q., Filling Mesopores of Conductive Metal–Organic Frameworks with Cu Clusters for Selective Nitrate Reduction to Ammonia. *ACS Appl. Mater. Interfaces* **2022,** *14*, 32176-

32182.
9. Wang, Z.; Liu, S.; Wang, M.; Zhang, L.; Jiang, Y.; Qian, T.; Xiong, J.; Yang, C.; Yan, C., In Situ Construction of Metal–Organic Frameworks as Smart Channels for the Effective Electrocatalytic Reduction of Nitrate at Ultralow Concentrations to Ammonia. *ACS Catal.* **2023,** *13*, 9125-9135.
10. Sun, W.-J.; Ji, H.-Q.; Li, L.-X.; Zhang, H.-Y.; Wang, Z.-K.; He, J.-H.; Lu, J.-M., Built-in Electric Field Triggered Interfacial Accumulation Effect for Efficient Nitrate Removal at Ultra-Low Concentration and Electroreduction to Ammonia. *Angew. Chem. Int. Ed.* **2021,** *60*, 22933-22939.
11. Song, Z.; Liu, Y.; Zhong, Y.; Guo, Q.; Zeng, J.; Geng, Z., Efficient Electroreduction of Nitrate into Ammonia at Ultralow Concentrations via an Enrichment Effect. *Adv. Mater.* **2022,** *34*, 2204306.
12. Gong, Z.; Zhong, W.; He, Z.; Liu, Q.; Chen, H.; Zhou, D.; Zhang, N.; Kang, X.; Chen, Y., Regulating Surface Oxygen Species on Copper (I) Oxides *via* Plasma Treatment for Effective Reduction of Nitrate to Ammonia. *Appl. Catal., B* **2022,** *305*, 121021.
13. Li, C.; Liu, S.; Xu, Y.; Ren, T.; Guo, Y.; Wang, Z.; Li, X.; Wang, L.; Wang, H., Controllable Reconstruction of Copper Nanowires into Nanotubes for Efficient Electrocatalytic Nitrate Conversion into Ammonia. *Nanoscale* **2022,** *14*, 12332-12338.
14. Xu, Y.; Wang, M.; Ren, K.; Ren, T.; Liu, M.; Wang, Z.; Li, X.; Wang, L.; Wang, H., Atomic Defects in Pothole-Rich Two-Dimensional Copper Nanoplates Triggering Enhanced Electrocatalytic Selective Nitrate-to-Ammonia Transformation. *J. Mater. Chem. A* **2021,** *9*, 16411-16417.
15. Ren, T.; Ren, K.; Wang, M.; Liu, M.; Wang, Z.; Wang, H.; Li, X.; Wang, L.; Xu, Y., Concave-Convex Surface Oxide Layers over Copper Nanowires Boost Electrochemical Nitrate-to-Ammonia Conversion. *Chem. Eng. J.* **2021,** *426*, 130759.
